# Zooarchaeology of the social and economic upheavals in the Late Antique-Early Islamic sequence of the Negev Desert

**DOI:** 10.1038/s41598-019-43169-8

**Published:** 2019-04-30

**Authors:** Nimrod Marom, Meirav Meiri, Yotam Tepper, Tali Erickson-Gini, Hagar Reshef, Lior Weissbrod, Guy Bar-Oz

**Affiliations:** 1 Leon Recanati Institute for Maritime Studies, University of Haifa, Haifa, Israel; 2 Steinhardt Museum of Natural History, Tel Aviv University, Tel Aviv, Israel; 3 Israel Antiquities Authority, Jerusalem, Israel; 4 Department of Archaeology, University of Haifa, Haifa, Israel; 5 Zinman Institute of Archaeology, University of Haifa, Haifa, Israel

**Keywords:** Climate-change ecology, Zoology

## Abstract

The Byzantine – Islamic transition (7^th^–8^th^ centuries CE) in the desert-edge Palaestina Tertia is examined using faunal remains recovered from archaeological sites in the Negev. Archaeozoological analyses suggest sharp differences between Late Byzantine and Early Islamic animal economies, especially in herding patterns and the exploitation of wildlife resources. These differences are suggested to reflect both cultural and land ownership changes following the Arab conquest, against the backdrop of climatic change. The archaeozoological record thereby provides independent evidence to the rise and fall of societal complexity in this marginal region.

## Introduction

The Negev Desert between the 4^th^ and 8^th^ c. CE witnessed the local reverberations of dramatic historical events of the Late Roman, Late Antique (Byzantine), and Early Islamic periods, spanning the rise and fall of the Roman Empire and emergence of Christianity and Islam. At the same time, human communities in this arid and marginal region of the southern Levant variably negotiated unique challenges to the development of settlement, agriculture and trade, against the background of climatic change^1–3^. Archaeologists have long aimed to employ the material record of the Negev in order to integrate knowledge of broad historical dynamics with their manifestations in regional and local sequences^4–15^. An especially controversial issue centers on explanations of the exceptional fluorescence and subsequent decline of Byzantine-period settlement in the Negev, with researchers differentially drawing on sources of evidence from the historical, material and paleoclimatic records to construct alternative narratives^7,16–23^. Our view of the internal history of the Negev during this crucial period and the way it was impacted by external political, economic and climatic forces remains, however, incomplete: archaeozoological evidences to environmental and cultural change have not received integrative analytic consideration to date.

The expansion of settlement in the Negev beginning in the Late Roman and Early Byzantine periods (4^th^–5^th^ c. CE), which reached a climax in the Middle Byzantine 5^th^ c. CE, marked a singular event in the history of this marginal region^2^. Through much of the late Holocene, human occupation of the arid Negev involved the presence of nomadic pastoralists, short-term military stations, isolated small-scale farmsteads and trading posts^7,16,22^. This began to change with the Romanization and systemic settling down of Nabataean nomadic trading tribes from the 2^nd^ c. CE^17^. From the 4^th^ c. CE large-scale expansion of sedentary settlements and agricultural activity in the Negev went together with the establishment of the Byzantine Empire^10,13,14^.

Intensive farming, which was the foundation for the Byzantine settlement in the arid Negev, relied on the development of sophisticated methods for water conservation and harvesting, soil fertilization, and adaptive management of crop and livestock regimes^6,8,22,23^, and also, arguably, on a short-lived increase in rainfall^24^. A vivid expression of the success of Negev agricultural development by the Byzantines is the international distribution and reputation of locally produced wine^23,25,26^. Although famous for its wine, the florescent Byzantine agriculture must have been integrated with animal resource management systems. Here we report, for the first time, an analysis of animal resource exploitation in the Negev, and use it to test common assertions regarding shifting social dynamics among settled and nomadic groups, land use practices, trade and foodways.

## Models of Livestock Management

Models of livestock production and provisioning in systems of nomadic and settled societies predict variable herd management strategies, depending in large part on relations of production in settler-nomad social systems and availability of resources in arid environments^27–31^. By “nomad” we mean specialized livestock herders, who are not connected socially and economically to a specific settlement, and who practice long-range transhumance. Sheep (*Ovis aries*) and goat (*Capra hircus*) herding is generally widespread among mobile pastoralists of the Negev as shown by a range of archaeological and ethnographic studies in this region^32–36^. A preference for sheep over goats has been observed in contexts where access to extensive pastureland is relatively unrestricted and production is oriented to urban markets^32,36,37^. In these contexts, specialized producers maintain close relations of exchange with settled consumers who are typically engaged in non-farming activities (e.g., trade, military). Such settled communities would also consume more sheep than goats, since their main source of livestock supplies would be specialized producers, namely nomads^30,31^. In contrast, under conditions of sedentary, mixed agropastoral practices we would expect to see less farmer-herder labor division and an increasing preference for goats over sheep. The reason for the preference for goats within sedentary farmers’ strategy of minimizing loss in a worst case scenario is the ability of goats to subsist on degraded, over-grazed pasture^36^, and also on their high resilience to disease and draught, combined with a high birth rate^38,39^. Water shortages in the arid Negev could have limited the use of more water-dependent livestock species (e.g., cattle, *Bos taurus*), while changing cultural and religious orientations, including the introduction of Christianity and, later, of Islam, could have imposed different sanctions on the consumption of pig (*Sus scrofa*)^40,41^.

Because of the intimate association between livestock management and social organization, more comprehensive understanding of the cultural trajectory of the Negev is made possible by considering the faunal evidence from local sites. The faunal data greatly complement and extend what we know presently from the artefactual record by providing direct evidence on human-environment interactions and the way that these played out through daily social and economic transactions. In this paper we present the first study of a long-term faunal sequence obtained in recent campaigns in four key sites of the Negev, covering the Late Antique-Early Islamic period (Avdat, Shivta, Halutza, and Nessana). This study also uniquely focuses mainly on material obtained from ancient garbage deposits, found both inside and outside of the Negev settlements and shown to contain especially rich concentrations of archaeozoological remains. Employing such a longitudinal approach with a high temporal resolution backed by carbon 14 dating, we show how societies reacted locally to the vagaries of regional politics and the environment while accounting for both the opportunities and limitations of their surroundings. Archaeozoological assemblages chosen for this analysis represent key phases of settlement expansion in the Byzantine and decline in the Early Islamic periods through the Negev sequence (Table 1).

**Table 1:** Contexts and sample sizes in relation to expected duration of accumulation of the faunal assemblages reported in this study.

Assemblage	Context	Date	Sample size	Duration	Analytical contribution
Avdat	Midden under earthquake collapse in domestic quarters	2nd — Late 4th century CE	486	Long term	Consumption discard in military-affiliated domestic quarters of an agricultural and local trade center; Romanized Nabataeans.
Halutza	Two garbage middens outside the town	6th -early 7th centuries CE	443	Long term	General patterns of animal economy in the Byzantine period settlement. Christian population.
Shivta	Two garbage middens outside the town	Late 6th -early 7th centuries CE	143	Long term	General patterns of animal economy in the Late Byzantine period agricultural settlement. Christian population.
	Two garbage middens inside the town	7th–8th centuries CE	84	Long term	General patterns of animal economy in an Early Islamic settlement. Muslim or mixed Muslim/Christian population.
Nessana	Garbage middes around the town	Late 6th -early 7th centuries CE	141	Long term	General patterns of animal economy in the Late Byzantine period agricultural settlement
	Garbage midden inside the town	Late 7th–early 8th centuries CE	394	Short term	Pre-abandonment deposits of a Byzantine settlement

On top of our primary interest in diachronic change, we pay careful attention to the internal complexity of the Negev settlement system, including differences in the character of human occupation in each of the study sites. In two of the sites, Nessana and Shivta, we incorporate assemblages from two different periods so that within-site variation through time can also be considered. This has special bearing on persisting issues in understanding the nature of the transition between the Byzantine and Early Islamic periods—whether it involved mainly continuity or break in local cultural and economic traditions. The diachronic analysis of the fauna may advance our understanding of the reasons for the eventual decline in permanent settlement in the Negev, and its ultimate abandonment lasting until the modern era.

## Sites and Setting

**Figure 1:**
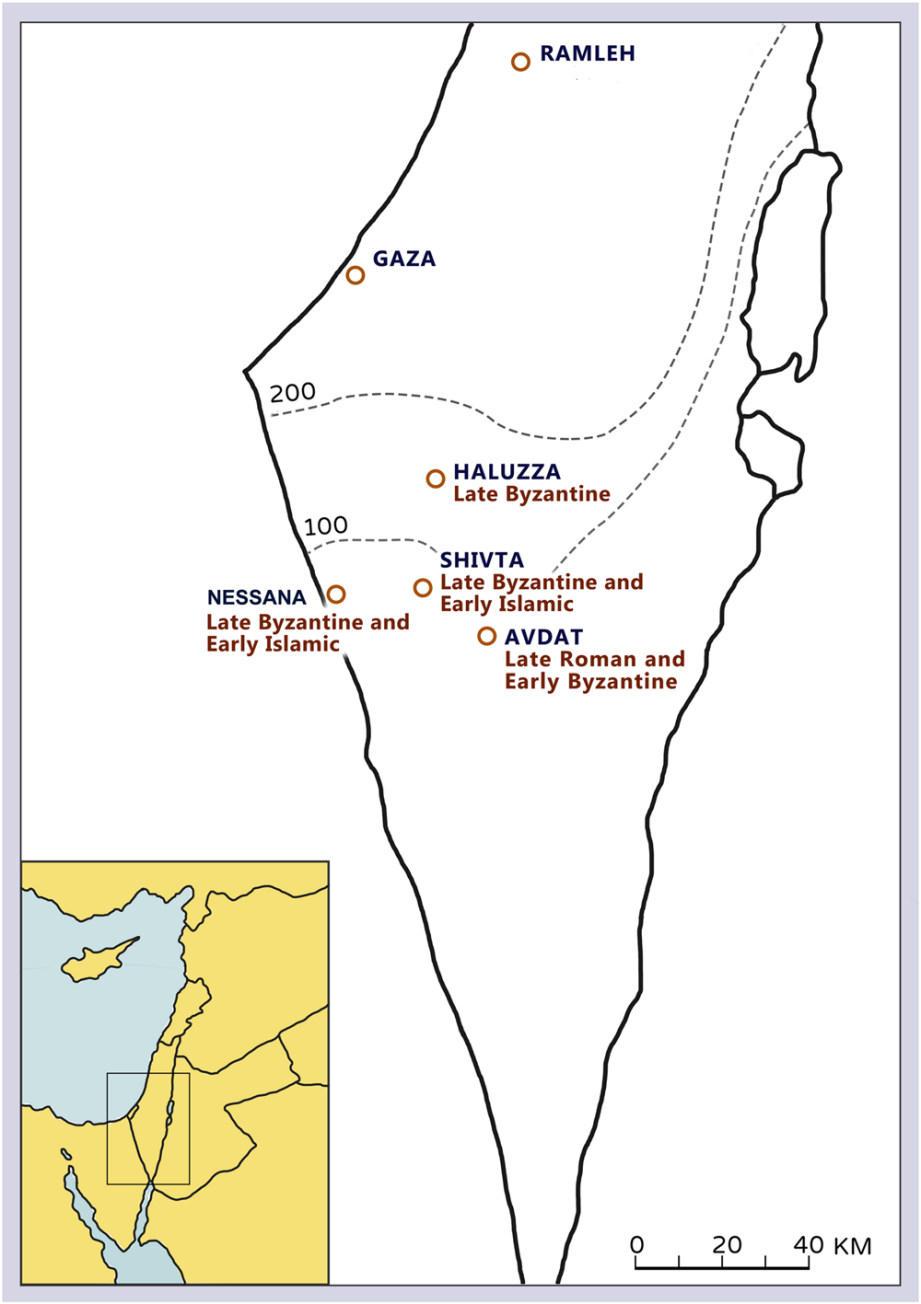
Location map for the sites mentioned in the text. Map drawing by Aya Mark.

The assemblages described in this study were excavated in the central part of the Negev, a desert region in southern Israel. The Negev grades from semi-arid conditions in the North and West to arid conditions in the South and the East, with the excavated sites located in a region which today receives 100 mm mean annual precipitation (Fig. 1). Changes in the Negev settlement pattern and intensity have taken place against the background of regional climatic shifts^42^, with wetter Byzantine 6^th^ and early 7^th^ centuries bracketed by dry conditions in the late Roman (4^th^–5^th^ centuries CE) and the Early Islamic (later 7^th^–10^th^ centuries CE) periods^43^.

These shifts comprise of climatic fluctuations of uncertain tempo and punctuality superimposed on a late Holocene drying trend. It is not clear that such climatic shifts significantly affected the arid Negev in Antiquity, but there seems to be a consensus that the Byzantine period enjoyed a somewhat wetter environment, followed by a shift to arid conditions, like the present, toward the later part of the first millennium CE at the latest^44,45^ as part of a global rapid climate change event^46^. Regional evidences to such post-Byzantine aridification comes to date from sedimentological studies on fluvial activity that reached a peak in the Byzantine period^47,48^, changes in the Dead Sea pollen^49^ and water levels^24,50^, which fall at the early seventh century with a magnitude indicative of a sharp climatic event^51^. Ardification at the late first millennium CE is also inferred from pollen studies in Makhtesh Ramon^52^ (see ref.^52^: Fig. 4) and paleo-wetlands around the Dead Sea^51^ showing a decline in grasses. Other evidences to late first millennium aridification include cessation of west Negev dune formation suggesting a changing wind regime^45^, high-resolution speleothem analysis from Mediterranean Soreq Cave showing a drying trend unto the end of the 1st millennium CE^53^, and historical correspondences from the Nessana archives indicating episodes of draught^54^. To these one might add the cessation of the use of the southern pastures around Makhtesh Ramon following the Early Islamic period^55^.

Our analysis includes faunal assemblages from the following sites

### Avdat

In late 1999 and early 2000 a large-scale excavation was carried out at the Nabataean Roman site of Avdat in the Negev Highlands by TEG on behalf of the Israel Antiquities Authority. The excavation focused on an area immediately outside and east of the Byzantine town wall, opposite the acropolis. It revealed several sequential dwellings including four rooms of one large Nabataean building constructed in the first century CE, a Roman Nabataean house with a stairway leading to an upper story dated to the late second through early third centuries CE, and several residential buildings constructed in the late third century CE that continued to be occupied until their destruction by earthquake in the early fifth century CE. The faunal assemblages from these different phases were pooled and are interpreted as representing consumption in a Romanized community of Nabataean descent, which subsisted from the Late Roman period onwards on agriculture and trade^56^.

### Haluza

In 2015 an excavation conducted by GBO, LW and TEG on behalf of Zinman Institute of Archaeology at the University of Haifa concentrated on the trash mounds on the periphery of the Byzantine town. Four squares were dug in two excavation areas, located on middens north and south of the town. The accumulated garbage in the middens is from the 5th — early 6th centuries CE and represents organized discard of urban consumption waste in an agricultural and administrative Christian community of the Late Byzantine period. The organized discard of garbage to the extra-mural middens ceased in the mid-6th century, more than a century before the onset of the Early Islamic period in the mid-7th century CE, indicating a change in settlement social organization and/or economy affecting the settlement before the Byzantine/Islamic transition.

### Shivta

In 2016 YT and GBO conducted an excavation in the village of Shivta. Trenches were dug in 20 different areas at the site. The excavations indicate that the settlement was inhabited already during the later Roman period, although few remains from that period were found. Most of the archaeological finds has been recovered from Late Byzantine 5th — early 7th landfills on the periphery of the village, representing organized garbage management as in Halutza. Fewer finds were collected from the Early Islamic mid-7th — 8th centuries, mainly from household middens that accumulated inside many deserted buildings from the Late Byzantine fluorescence of the town. The population in the Early Islamic period was probably Muslim, as evidenced by an inscription- and cross-bearing lintel stone from a great Byzantine church as a stepping stone at the entrance to a building^23^. Permanent settlement at Shivta ceased in the 8th century CE.

### Nessana

In 2017 YT, GBO, LW and TEG directed an excavation in the town of Nessana, where previous archaeological work^4^ revealed domestic quarters, churches, and an archive of papyri from the 6th–7th centuries CE^57^. The new excavations were conducted in six areas in the lower part of the settlement, which comprised of both domestic contexts and garbage middens. As in Shivta and Halutza, garbage from the Byzantine period was discarded outside the peripheral wall of the settlement, representing consumption in a Christian, agricultural community. Garbage from the Early Islamic period was dumped inside the precincts of the old town wall, which was already out of use, and inside abandoned Byzantine buildings. The archaeozoological finds presented here were obtained mainly from this later phase, which represents the last years of consumption and discard activity at a neighborhood that housed an ecclesiastical community^58^.

## Methods

Faunal remains of key livestock taxa including mainly sheep, goats, cattle, and pigs were retrieved through on-site hand collection from excavations in the sites of Avdat, Shivta, Nessana and Halutza. Additional systematic collection of remains of smaller species was conducted through dry-sieving of all excavated sediments through a 5 mm mesh in Shivta, Nessana and Halutza. This study is concerned mainly with remains of the large fauna, found almost exclusively in the hand-collected fraction. The analyzed material is stored in the Laboratory of Archaeozoology, University of Haifa to be transferred for long-range storage in 2020 to the Israel Antiquity Authority.

**Figure 2:**
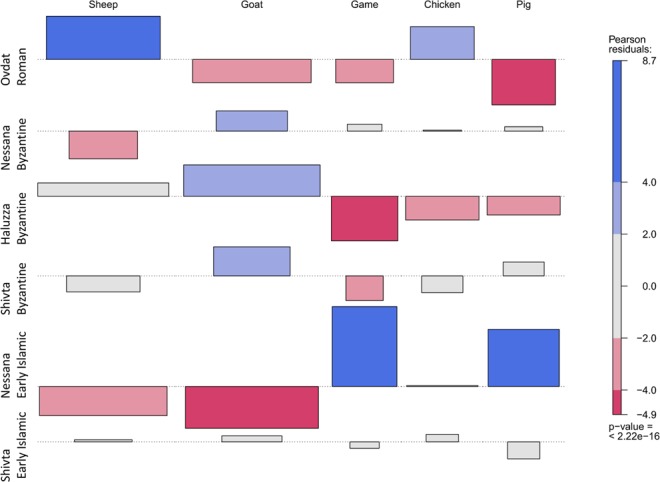
Association plot of the taxonomic composition of the faunal assemblages from the Negev. Bin sizes correspond to sample size; Pearson residues showing significant positive (blue) or negative (red) departure from expected cell values. Grey cells indicate no departure from expected values. Silhouettes indicate the over-/under-represented taxonomic categories. Row and column names appear in the margin. OVD_ROM = Late Roman — Early Byzantine Avdat; NIZ_BYZ = Late Byzantine Nessana; HAL_BYZ = Late Byzantine Halutza; SHIV_BYZ = Late Byzantine Shivta; NIZ_EIS = Early Islamic Nessana; SHIV_EIS = Early Islamic Shivta.

The faunal remains were identified using the comparative collection of the Laboratory of Archaeozoology at the University of Haifa by one of us (NM). Caprines (sheep, *Ovis aries*, and goats, *Capra hircus*) were distinguished based on morphological and metric criteria^59^. Geometric morphometric analysis of two pig lower third molars from Nessana was used to determine if they are of wild or domestic individuals (Supplementary S1 Figs 1, 2), and identification of three rare hartebeest specimens was confirmed using ancient mtDNA analysis by MM in the Laboratory of Ancient DNA, Tel Aviv University (Supplementary Table S2). To obtain accurate quantitative estimates of species composition and of body-part spectra across the different assemblages we used the identification method of diagnostic zones^60–62^. Data on the state of epiphyseal fusion^63^, tooth eruption and wear^64^ and osteometric measurements^65^ (Supplementary Table S3) were also collected. Bone fracture morphology^66^ and surface modifications such as gnawing, weathering and butchery were noted for all identified fragments. Finally, statistical analyses were carried out with TPSdig^67^, and the vcd^68,69^, geomorph 3.0.6^70^ and ggtern 2.2.1^71^ packages for R 3.4.1^72^.

## Results

### Assemblage preservation

The assemblages obtained from stratified deposits in light, dry soils of the Negev are all well preserved, as shown by taphonomic observations (Supplementary Table S4). The sample sizes in some of the assemblages studied are small, especially EIS Shivta. The frequency of weathered specimens is low (<5%), with a nearly pristine state of bone cortical surfaces on most specimens. This suggests rapid burial and buildup of garbage deposits. Bone surface modifications generally comprise of a low frequency (<10%) of burned and gnawed specimens, cut- and chop- marks. Fragmentation was extensive, however, and the presence of high frequencies of fractures on both dry and fresh bones suggest that bone breakage was caused during both food preparation and through post-depositional diagenetic processes^73^ (Marom 2016). All major livestock taxa show high skeletal completeness and representation of different parts of the animal body, with body-part profiles that are not biased towards any specific element (Supplementary Table S5). This observation is consistent with a process of animal disarticulation, consumption and discard of complete livestock carcasses occurring on site. In a few rare taxa of transport animals or wild game only a few of the skeletal elements are present, parsimoniously explained by low sample sizes.

### Livestock species

The studied assemblages are dominated by the remains of livestock, especially caprines (Table 2). Clear changes in the frequency of different species of livestock can be observed between periods (chi-squared = 309.78, p-value = 0.0004, N_resample_ = 2000; standardized Pearson residuals appear in Fig. 2; sheep/goat ratios were first determined in the fraction of caprine remains identifiable to species then projected on the entire caprine sample: for sample sizes see Table 2). Our results show that in Late Roman — Early Byzantine Avdat, sheep are by far the most dominant taxon occurring with fewer goats and only rare remains of pig. Chicken are common in this assemblage, and wild game are rare. Three assemblages, however, from the Late Byzantine period in Shivta, Nessana and Halutza show the dominance of goats over sheep. Here there are increased frequencies of pigs and game animals compared to the earlier Avdat assemblage. In Early Islamic Shivta, the frequency of pigs is comparatively low, and sheep and goats occur in similar numbers. These livestock animals, with some chickens, appear to have comprised the mainstay of the 8^th^ century village economy.

**Table 2:** Number of identified specimen counts.

Taxon	Nessana				Halutza		Avdat		Shivta				Total
	Late Byzantine		Early Islamic		Early-Middle Byzantine		Late Roman – Early Byzantine		Late Byzantine		Early Islamic		NISP
	N	%	N	%	N	%	N	%	N	%	N	%	
Sheep	9		29		44		125		9		6		222
Goat	30		26		55		66		20		7		204
Sh/G	60	70	141	50	288	87	196	80	84	79	50	75	819
Pig	11	8	69	18	23	5	1	*	16	11	2	3	122
Cattle	3	2	6	2	7	1	10	2	1	*			27
Camel	1	*					18	4	1	*	1	1	21
Equid	3	2	4	1	1	*	13	3	1	*			22
Chicken	10	7	34	9	20	5	45	9	5	3	9	11	123
Pigeon	3	2	3	1	1	*	1	*	2	*	1	1	11
Hartebeest	2	1	3	1	1	*	9	2					15
Gazelle	8	6	70	18	2	*	1	*	1	*	4	5	86
Hare			4	1	1	*					1	1	6
Duck			1	*			1	*					2
Reptile	1	*	4	1					3	2	3	4	11
Total	141		394		443		486		143		84		1691

Sh/G = sheep/goat. * = less than one percent.

Although sheep and goat dominate the livestock economies in the Negev during the period in question, other species as cattle, camels and equids are present in the faunal assemblages. Their combined frequencies are quite low, however (<5%). The paucity of cattle likely reflects the difficulty of keeping animals with high water requirements in an arid desert climate. Though equids and camels were kept in the Negev as evidenced by the presence of stables and information from historical texts^20^, their absence from garbage deposits in the studied settlements likely means that they were not part of the local diet. Chickens are present in low frequencies (<10%) in all sites, presumably providing a secondary source of meat and eggs^74,75^.

**Figure 3:**
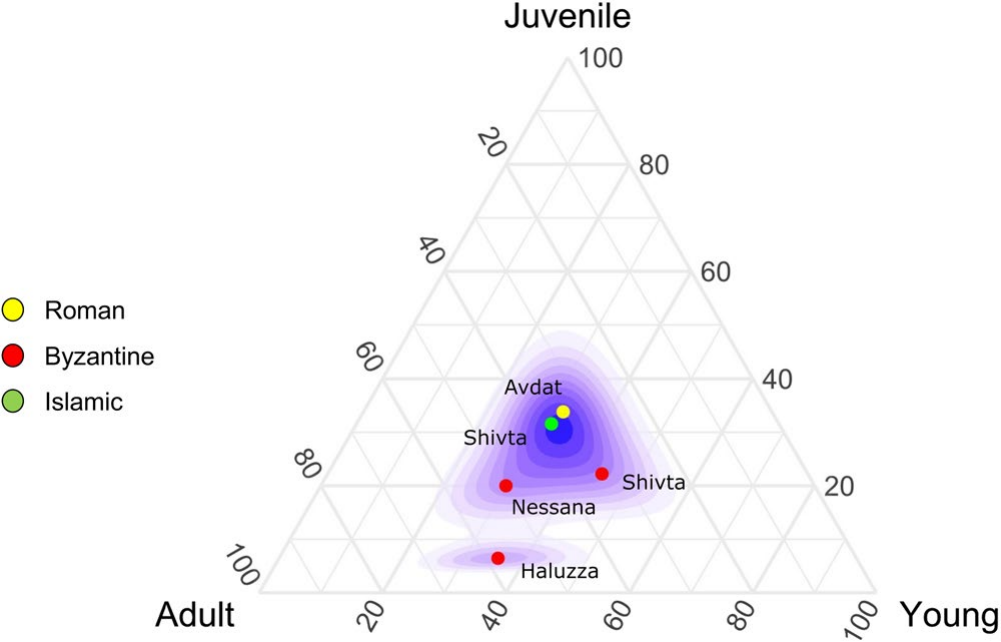
Ternary plot of caprine age-at-death based on tooth wear data (Table S7). Ages were pooled to juvenile (younger than one year at death), young (2–3 years at death), and older. Gaussian kernel density estimate for the underlying age distribution is marked by the red lines.

An analysis of the tooth eruption and wear-based age-at-death data for caprines shows two distinguishable groups (Fig. 3; tooth wear data in Supplementary Table S7): one group containing Late Byzantine assemblages of Shivta, Nessana and Haluza, and a second with Late Roman — Early Byzantine Avdat and Early Islamic Nessana. The Late Byzantine sites are characterized by a lower frequency (12%, N = 50) of juvenile caprines (2–3 years old) than in the Roman-Early Byzantine (34%, N = 68) and Early Islamic (32%, N = 19) assemblages. We view this more attritive mortality pattern in the Byzantine period as representing non-specialized production, as opposed to the market-age dominated samples from the Roman-Early Byzantine and Early Islamic periods.

### Wild animals

**Figure 4:**
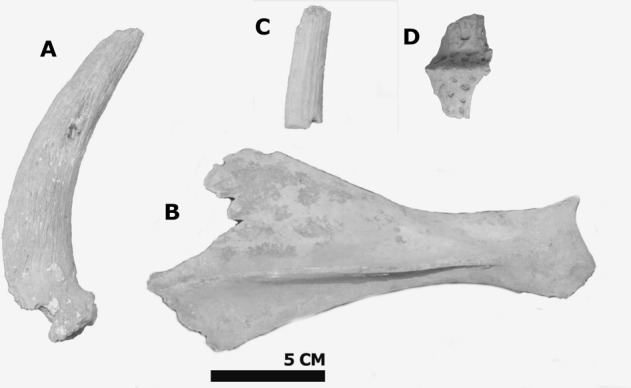
Clockwise from left: (**A**) mountain gazelle (*Gazella gazella*) horncore, Early Islamic Nessana, 105/1236-1; (**B**) hartebeest (*Alcelaphus buselaphus*) scapula, Nessana, 109/1283. (**C**) dorcas gazelle (*Gazella dorcas*) horncore fragment, Early Islamic Nessana, 107/1295; (**D**) crocodile dermal plate, Late Byzantine Shivta, 206/2028; Photography by NM.

Game species are represented mainly by gazelles (Fig. 4). In the Early Islamic assemblage of Nessana four out of five gazelle horncores belong to the local desert species, *Gazella dorcas*, and one horn core fragment to the Mediterranean Mountain Gazelle, *Gazella gazella*.

Two fragments of pig lower third molars from Early Islamic Nessana were compared to a Byzantine domestic pig population, to recent northern Israeli wild boar, and to the isolated wild boar population of the southern Dead Sea using geometric morphometrics (Supplementary S1 Figs 1, 2). A comparison of centroid size measures suggest that the Nessana specimens were similar in size to recent Israeli wild boar from the north, and are larger than both the domestic Byzantine pig sample and from recent Israeli wild boar from the Dead Sea region. A shape PCA on the Procrustes-fitted landmark data yields clear separation between the Byzantine domestic sample and the recent wild boar populations from both northern Israel and the Dead Sea region. The archaeological specimens from Nessana cluster with the wild boar sample on the first principle component, which explains 32% of the variability in shape. In summary, the geometric data suggest that the suids from Nessana were similar to northern Israeli wild boar in both size and shape. It is an open question if the suids represented by the teeth were hunted locally or traded in from the north.

Additional species include an antelope, that was identified to the species level as hartebeest (*Alcelaphus buselaphus*) based on ancient DNA. One sample from Halutza (GB24) (dated 540 CE) yielded 100% BLAST Maximum Identity with hartebeest (protocol and sample details appear in Supplement S2). The next species with the highest Maximum Identity after hartebeest are: Przewalski’s gazelle (*Procapra przewalskii*) found only in China, bighorn sheep (*Ovis canadensis*) found in North America and yellow-backed duiker (*Cephalophus silvicultor*) found in Central and Westren Africa. All these species had only 90% BLAST Maximum Identity. In summary, the ancient DNA data strongly suggest that the antelope from Halutza was a hartebeest, and not the morphologically-similar Arabian oryx. The sequence has been deposited in GenBank.

Hare (*Lepus capensis*), duck (*Anatidae* gen. et sp. indet), sea turtle (*Chelonia* sp.) and crocodile (*Crocodylinae* sp.)^1^,with the last two taxa clearly representing imports. The crocodile bone from Shivta (Locus 206, Basket 2028), a dermal plate (Fig. 2), is especially interesting as none were published to date from elsewhere in the Byzantine empire^76^.

Crocodiles, widely-worshipped in Egypt unto Roman times are reported to have been imported to Rome and kept in special pools with constructed basking platforms^77^. In Egypt itself, a late Roman parade armor made of crocodile skin was found in Manfalut (British Museum catalogue number EA5473), suggesting perhaps the adoption of local crocodile cults by legionnaires; a contemporary Egyptian text by Athenaeus, however, mentions roasted crocodile as “a most dainty dish”^78^, indicating that its use was also culinary. The presence in Shivta of a dermal plate rather than a meat-bearing skeletal element may hint at the use of a skin, rather than meat of the animal. Its possible meaning cannot be ascertained and could have ranged from an exotic/expensive status marker to ritual paraphernalia.

Hunting of occasional gazelles and hartebeest can be understood in terms of both sportive and subsistence activity (Marom and Bar-Oz 2013). Many of the gazelles are young, represented by osteologically immature specimens (19%, N = 35; based on epiphyseal fusion data in Supplementary Table S6). Although this proportion is lower than the that of juveniles in extant gazelle populations^79^ (~35%), it departs from documented patterns of archaeological low-intensity gazelle hunting and is on par with data from populations suffering from high levels of hunting pressure in the terminal Pleistocene^80,81^.

## Discussion

Our study of faunal remains from the Negev reveals the rise and fall of a sedentary agricultural economy in response to an amalgam of shifting social and environmental circumstances in the Byzantine-Early Islamic sequence. A goat-based pastoral economy (GPE) emerged for a relatively short temporal span during the height of the Byzantine period (5^th^–6^th^ c. CE) replacing a long stable pattern of a sheep-based pastoral economy (SPE) endemic to nomadic Negev herders throughout much of the region’s history until recent times. Additional evidence for these economic transitions comes from data on the age composition of caprine assemblages from the study sites. The Byzantine GPE-type assemblages are also characterized by relatively low frequencies of juvenile individuals, contrasting with more even representation of different age classes as seen before and after this time span. This pattern accords with a generalized strategy of livestock production associated with a sedentary way of life as opposed to more specialized sheep pastoralism, which depend on access to markets for their surpluses as well as requiring more pasture land, water and better quality graze.

The long standing emphasis on sheep within an SPE oriented strategy is a near universal pattern of specialized livestock production among nomadic pastoralists in a marginal environment^82,83^. In such environmental contexts agricultural production is usually neither viable nor profitable. This SPE pattern has been documented in the Negev during different periods and is considered a durable feature of non-agricultural settlements such as forts^34^ and trade outposts^84^. It fits well with models of livestock production in pastoral economies oriented to supplying outside consumers and markets, also in the context of uninhibited access to pasture^30,31^. Negev Bedouins in the 19th century tended to herd caprine herds in such open pastures, maximizing the sheep-goat ratio whenever possible^85^. The near absence of pigs or cattle in such contexts suggests a resilient strategy of local inhabitants to arid and challenging conditions in the region. In Early Byzantine and preceding Roman and Hellenistic periods such economic strategies would have been associated with the presence of nomadic pastoralists, conventionally labelled “Nabateans”.

We argue for a broadly occurring transition to a GPE strategy of preference for goats over sheep during a restricted period in the Negev’s history in the Early-Middle Byzantine. This transition, which we observe in three different sites with Byzantine assemblages, indicates the establishment of settled agricultural communities where at least a part of the meat supply is grown and consumed locally^30,36^. Likewise, settled communities of Sinai Bedouins in recent times have tended to keep more goats than sheep (GPE) with the former adapted to overgrazed pasture near permanent settlements^36^, and offering sedentising herders added advantages of greater drought and disease resistance. GPE strategies are advantageous where consumption is mainly local rather than market oriented, and mobility is limited. This contrasts with specialized pastoral nomads with an SPE strategy, which target higher market value for their products in times when markets are available, but depend on access to extensive pasture grounds^30,32,33^. Unlike with SPE production strategies for market trade, settled farmers pursuing a GPE strategy are also able to take advantage of the reduced mortality rates of goats compared to those of sheep in the less flexible and more stressed conditions of sedentary communities^30,86^. In simple terms, sheep husbandry (SPE), which has prevailed in the Negev for much of its history, requires more human labor, water and land resources and hence competes with agricultural production in sedentary communities^87^ (Schneider’s^88^ “Sheep eat men” idiom). Our data suggest that this otherwise resilient strategy among nomadic and flexible herders became temporarily obsolete in the main hubs of settlement of the Negev with the settling down and shifting economic focus of Byzantine communities.

A second economic turnaround in the record marks the end of a relatively short-lived Byzantine “experiment” with permanent settlement and agricultural development in the Negev. This transition is observed most clearly in our sequence in the data from a single site. In Shivta, goats are significantly overrepresented in Byzantine period garbage deposits (GPE), whereas material from the Early Islamic period shows the most equitable composition of livestock species in the entire sequence (i.e., neither a GPE or SPE pattern). Similarly, in the Early Islamic assemblage of the nearby site of Nessana sheep and goat are present in comparable proportions, though both are significantly underrepresented relative to other species in this assemblage under a hypothesis of homogeneity with the composition of other assemblages. It is of note that the underrepresentation of goats in this case is more pronounced compared to that of sheep; the frequency of both taxa decreases, but more so of goats.

In the Early Islamic period of Nessana we see a sharp departure from the caprine dominated assemblages of all preceding periods in our sequence. Unlike in any of the other assemblages in this site suids, suggested to have been wild boar hunted locally or imported, and wild antelopes (gazelle and hartebeest) comprise the dominant part of the faunal assemblages with significant overrepresentation in the numbers of both taxa. In a contemporary assemblage from Shivta pig frequencies are considerably lower, possibly in relation with a cultural transition from a Romanized, Christian Byzantine community to an Islamic one^89^, also evidenced by the construction of a mosque at this time. Moreover, identification of two specimens of wild boar from Early Islamic Nessana suggests that meat of this species was hunted and consumed.

These idiosyncratic food preferences in Nessana go along with strong intensification in hunting activities, mainly targeting local populations of gazelle and possibly wild boar. The age profile of hunted gazelles is consistent with a population under severe human hunting^81^. Intensive hunting in the Negev may have brought about the final extinction of hartebeest, which was previously thought to have gone extinct at a considerably earlier time^90–92^. This highly atypical subsistence pattern incorporating substantial numbers of pigs, hunted gazelle and wild boar concords with the presence of a spatially-confined economy relying on wild resource exploitation and trade, and Christian rather than Islamic affiliation.

Such conditions could have developed during the land survey conducted in the late 7th century after the establishment of Arab control over the region. This survey that resulted in the allocation of land to Bedouins, who probably used it for pasture. Nessana Papyrus 58 contains a request for tax, to be paid by one Sergius to the governor at Gaza on land given to him for agriculture: the land was transferred to Sergius from an Arab group (Beni Wa’er)^93^. Transfer of ownership over pasture land to Arab tribes would also explain why sheep and goats are not mentioned at all in the Early Islamic Nessana Papyri^22^: sheep and goats were not husbanded by the Nessana community. The shift to pastoralism can potentially be observed in the ephemeral nomad settlements of the region, dated broadly by archaeological survey to the Late Byzantine/Early Islamic^16,22^. If the pastoral-Bedouin model suggested above is correct, future archeometric dating of these settlements would hypothetically show a clearly Early Islamic (rather than Byzantine) peak.

## Conclusion

The faunal remains from the Negev sequence presented above shows the hitherto unstudied aspect of the livestock management practices that accompanied the Byzantine agricultural florescence of the Negev. These practices focus on goats, which are adapted to sedentary, and therefore resource poor, pastural resource management. This focus on a goat dominated pastoral system stands in contrast to earlier and later desert societies, that endorsed mobility and sheep-based pastoral systems. Additionally, the faunal remains from the dumps of the last Christian community at Nessana suggests a phase of limited access to resources needed for pastoral production. In this last throw of the Byzantine Negev, the Christian community at the site has recourse to intensive exploitation of wild game resources.

## Supplementary information

Supplements
