# Supplementary material for: Zooarchaeology of the social and economic upheavals in the Late Antique-Early Islamic sequence of the Negev Desert
Source: Sci Rep. 2019 Apr 30;9:6702. doi: 10.1038/s41598-019-43169-8 (PMC6491595; doi:10.1038/s41598-019-43169-8)
Supplement: Supplementary file 1 — Supplements [file 41598_2019_43169_MOESM1_ESM.pdf]

Zooarchaeology of the social and economic upheavals in the Late Antique-Early Islamic sequence of the Negev Desert

Nimrod Marom, Meirav Meiri, Yotam Tepper, Tali Erickson-Gini, Hagar Reshef, Lior Weissbrod, and Guy Bar-Oz

SUPPLEMENTARY MATERIALS

Supplement 1

Two partially-preserved pig teeth from Nessana were compared to recent populations of Israeli wild boar from the northern part of the country (N=10), from the Dead Sea region (N = 3), and a population of Byzantine domesticated pigs from a single site (N=19). 2D landmarks (N=7) and semisliding landmarks (N=60) were used to capture the shape of the part of the teeth preserved in the archaeological specimens from Nessana, --->

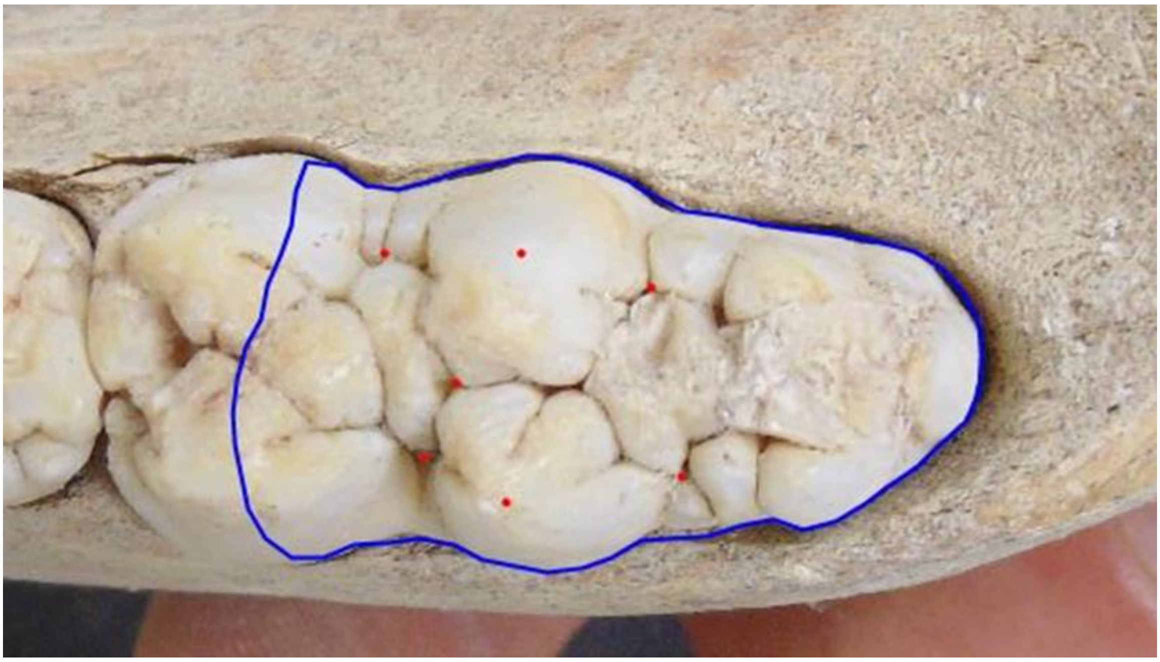

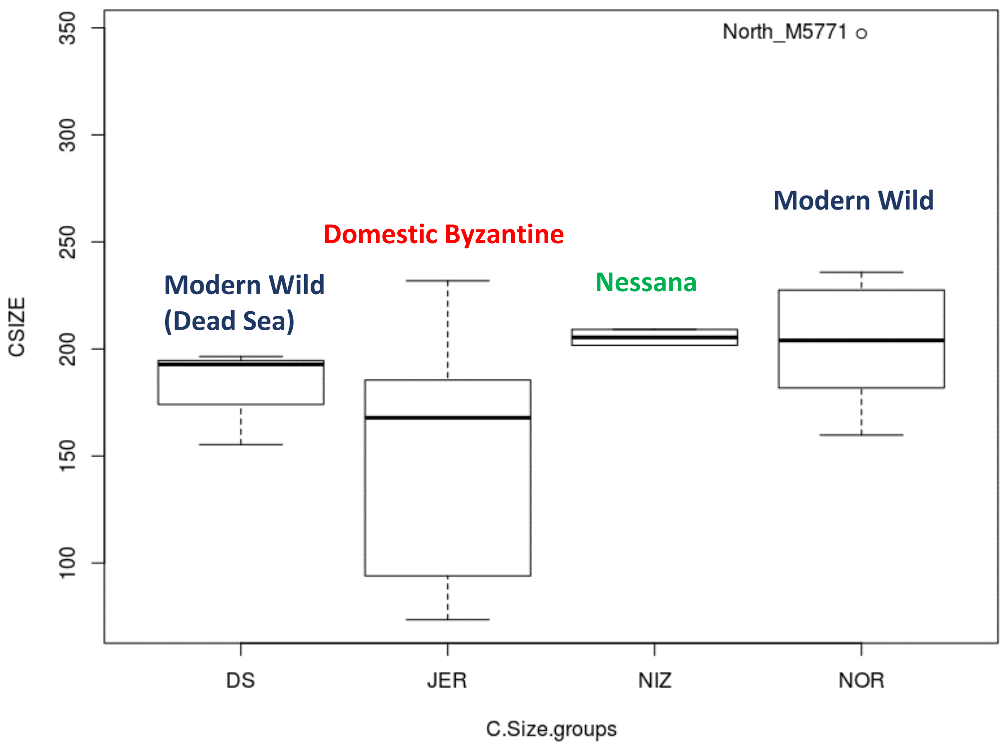

S1 Fig. 1 presents a box-and-whisker plot of the centroid size of the speciemens from the archaeological and recent samples, along with the shape ANOVA results (that exclude the small Nizzana sample). Photograph by NM.

```
 Df Sum Sq Mean Sq F value Pr(>F)
C.Size.groups  3  25906    8635    3.47 0.0287 *
Residuals     29  72175    2489
---
Signif. codes:  0 '***' 0.001 '**' 0.01 '*' 0.05 '.' 0.1 ' ' 1
```

LEGEND

DS	Dead Sea, IL	N=3
JER	Jerusalem, Byzantine	N=10
NIZ	Nessana	N=2
NOR	North, IL	N=9

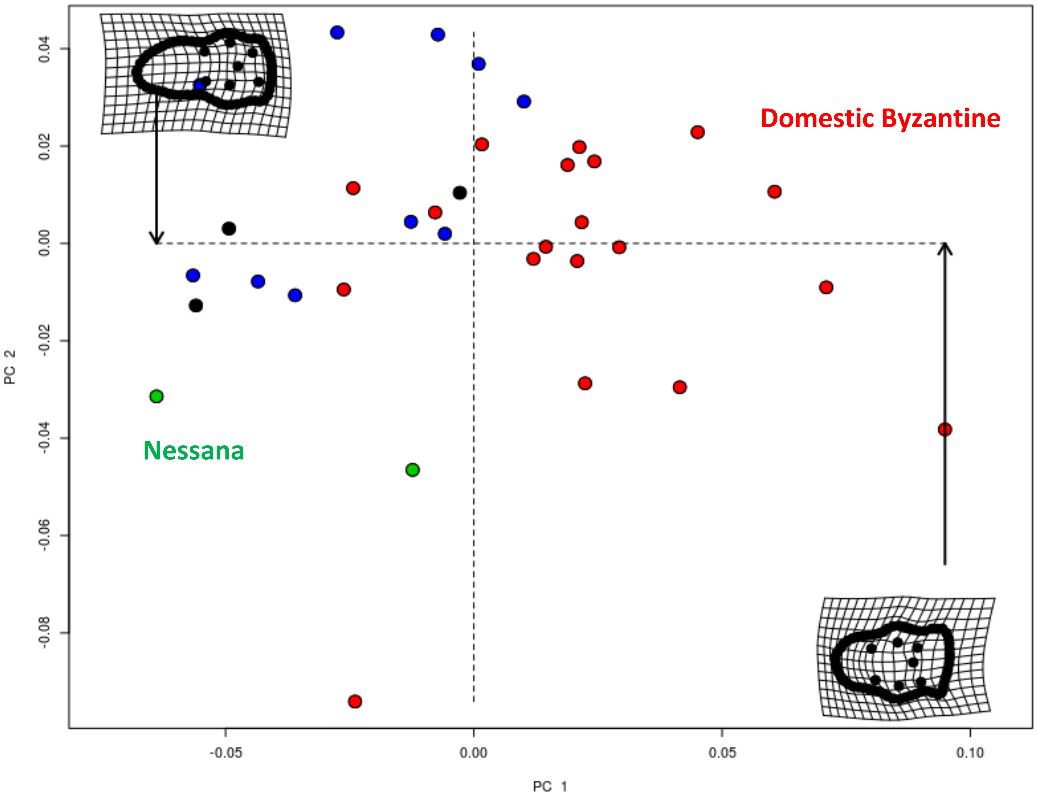

S1 Fig. 2 presents PC1 & PC2 of the shape PCA results, plotted by the plotTangentSpace function in Geomorph. The Byzantine domesticated sample from Jerusalem (red) plots mainly in Quadrant 1; recent wild boar from northern Israel (blue) and the Dead Sea region (black) plot mainly in Quadrant 2; and the two Nessana speciemens (green) in Quandrant 3.

		Sample	N
LEGEND	green=	Nizzana	2
	red=	Jerusalem, Byzantine domestic	19
	blue=	Recent, northern IL	10
	black=	Recent, Dead Sea region, IL	3

PC Summary

Importance of first k=33 (out of 34) components:

	PC1	PC2	PC3	PC4	PC5	PC6	PC7	PC8
Standard deviation	0.03803	0.02755	0.02412	0.01667	0.01513	0.01413	0.01271	0.01060
Proportion of Variance	0.32298	0.16943	0.12989	0.06206	0.05114	0.04458	0.03607	0.02509
Cumulative Proportion	0.32298	0.49241	0.62230	0.68436	0.73550	0.78008	0.81614	0.84124

	PC9	PC10	PC11	PC12	PC13	PC14	PC15
Standard deviation	0.009644	0.009057	0.008502	0.00797	0.007516	0.006814	0.006585
Proportion of Variance	0.020770	0.018310	0.016140	0.01418	0.012610	0.010370	0.009680
Cumulative Proportion	0.862010	0.880320	0.896460	0.91064	0.923260	0.933630	0.943310

	PC16	PC17	PC18	PC19	PC20	PC21	PC22
Standard deviation	0.006131	0.005798	0.00516	0.005091	0.004743	0.003888	0.003669
Proportion of Variance	0.008390	0.007510	0.00595	0.005790	0.005020	0.003370	0.003010
Cumulative Proportion	0.951700	0.959210	0.96515	0.970940	0.975970	0.979340	0.982350

	PC23	PC24	PC25	PC26	PC27	PC28	PC29
Standard deviation	0.003512	0.00339	0.00329	0.002957	0.002804	0.002538	0.002382
Proportion of Variance	0.002750	0.00257	0.00242	0.001950	0.001760	0.001440	0.001270
Cumulative Proportion	0.985100	0.98767	0.99008	0.992040	0.993790	0.995230	0.996500

	PC30	PC31	PC32	PC33
Standard deviation	0.00231	0.002032	0.002001	0.00149
Proportion of Variance	0.00119	0.000920	0.000890	0.00050
Cumulative Proportion	0.99769	0.998610	0.999500	1.00000

Type I (Sequential) Sums of Squares and Cross-products
Randomized Residual Permutation Procedure Used
1000 Permutations
ANOVA effect sizes and P-values based on empirical F distributions

	Df	SS	MS	Rsq	F	Z	Pr(>F)	
population	3	0.043545	0.0145152	0.29464	4.1772	4.6405	0.001	**
Residuals	30	0.104246	0.0034749	0.70536				
Total	33	0.147792						

Signif. codes: 0 ‘***’ 0.001 ‘**’ 0.01 ‘*’ 0.05 ‘.’ 0.1 ‘ ’ 1

Supplement 2

Lab #	Site	Type of Bone	Area	Square	Locus	Basket	Period
GB22	Haluza		B	6/01C	15/G-10		Byzantine
GB23	Haluza		B	6/01C	15/G-10		Byzantine
GB24	Haluza		B	6/01C	15/G-10		Byzantine

The DNA was extracted in a dedicated ancient DNA lab, at the Institute of Archaeology of Tel Aviv University, Israel, while the PCRs and post-PCR work were performed at the Zoology Department of Tel Aviv University, Israel. DNA extraction was according to a method modified from Yang *et al*. (1998) and described in Meiri *et al*. (2017). A very short fragment of the 3' end of the mitochondrial tRNA-Pro and 5' end of the control region was amplified with Hart1F (5'-CAAAGCTGAAGTTCTATTTAAACTA-3'), and Hart1R (5'- AAT TGA TAC TGA GAG GGC -3'). This very short fragment of 41bp was designed only to identify hartebeest as a species.

PCR amplifications were performed in 25ul reactions with: 1x PCR buffer, 1.5U of Platinum Taq DNA polymerase High Fidelity, 2 mM MgSO4, 0.2mM of each dNTP (all Invitrogen, UK) 0.1 mg/ml Rabbit Serum Albumin (Sigma-Aldrich Inc.), 0.4 µM of each primer (Sigma-Aldrich Inc.) and 2ul of DNA extract. The PCR amplification consisted of an initial denaturing at 94 ºC for four minutes, followed by 55 cycles of denaturing at 94 ºC for 30 sec, annealing at 50 ºC for 30 sec, and extension at 68 ºC for 40 sec with a final extension period of 5 minutes at 68 ºC.

The amplicons were cleaned from unincorporated primers using Exonuclease I, and Shrimp Alkaline Phosphatase (Thermo Fisher Scientific, UK). The samples were sequenced and analyzed in ABI 3100 Genetic Analyzer or Beckman-Coulter CEQT 8000 Genetic Analysis System. Sequencing was conducted on both strands.

The sequences were visually inspected and corrected using Geneious version 6.1 (created by Biomatters. http://www.geneious.com/), then aligned against sequence references (e.g. *Alcelaphus buselaphus*: Accession Number AJ235312; using BioEdit V7.2.5 (http://www.mbio.ncsu.edu/bioedit/bioedit.html). The sequences were blasted on GenBank (http://www.ncbi.nlm.nih.gov/genbank/), and species determined through % identity to the reported matches.

Meiri, M., Stockhammer, P.W., Marom, N., Bar-Oz, G., Sapir-Hen, L., Morgenstern, P., Macheridis, S., Rosen, B., Huchon, D., Maran, J., (2017). Eastern Mediterranean mobility in the Bronze and Early Iron Ages: inferences from ancient DNA of pigs and cattle, Sci Rep-Uk 7, 701.

Yang, D.Y., Eng, B., Waye, J.S., Dudar, J.C., Saunders, S.R. (1998). Technical note: Improved DNA extraction from ancient bones using silica-based spin columns, Am J Phys Anthropol 105, 539-543.

Supplement 3

Site	#	Locus	Basket	Period	Animal											
M3						L	W									
Haluza	G-10/15	1/03c	24	Byzantine	goat	25.4	8.8									
Haluza	G-10/15	1/03c	24	Byzantine	goat	25.7	10.5									
Haluza	G-10/15	1/03c	24	Byzantine	goat	26.2	9.8									
Nizzana	G4/17	113	1312/1	Byzantine	hartebeest	35	16.4									
Haluza	G-10/15	4/02c	18	Byzantine	sheep	22.8	8.4									
Haluza	G-10/15	1/04a	20	Byzantine	sheep	27	10.3									
Haluza	G-10/15	1/06a	33	Byzantine	shg	27.4	10									
Haluza	G-10/15	1/06a	33	Byzantine	shg	23.8	9.1									
Haluza	G-10/15	4/05d	48	Byzantine	shg		10									
Nizzana	G4/17	107	1295	Early Islamic	sheep	26										
Nizzana	G4/17	105	1236/1	Early Islamic	gazelle	15.7										
Nizzana	G4/17	121	1469/1	Byzantine	goat	24.1										
Nizzana	G4/17	103	1218/5	Early Islamic	goat	25.3										
Nizzana	G4/17	124	1418/1	Byzantine	pig	41.9										

Nizzana	G4/17	104	1267/2	Early Islamic	gazelle	16.8										
Haluza	G-10/15	1/05c	35	Byzantine	sheep	25.3	9.6									
Haluza	G-10/15	1/05c	35	Byzantine	sheep	28.2	10.8									
Haluza	G-10/15	1/03c	24	Byzantine	shg	22.7	9.7									
Ovdat	A-3141	1132	137	L Roman/ E Byzantine	donkey	30.7										
Ovdat	A-3141	704	95	L Roman/ E Byzantine	goat	27.8	11.2									
Ovdat	A-3141	"mezaveh"		L Roman/ E Byzantine	sheep	27.1	9.4									
Ovdat	A-3141	1202	43	L Roman/ E Byzantine	sheep		9.1									
Ovdat	A-3141	701-702	room 3	L Roman/ E Byzantine	sheep		9.5									
Ovdat	A-3141	702	42	L Roman/ E Byzantine	sheep		10.6									
Ovdat	A-3141	1122	126	L Roman/ E Byzantine	sheep	31.7	9.7									
Ovdat	A-3141	1122	126	L Roman/ E Byzantine	sheep	24.3	9									
Ovdat	A-3141	1143	171	L Roman/ E Byzantine	sheep	28	11.1									
Ovdat	A-3141	171	1143	L Roman/ E Byzantine	sheep	28.2										
Ovdat	A-3141	704	84	L Roman/ E Byzantine	sheep	28.5	10.7									
Ovdat	A-3141	1143	171	L Roman/ E Byzantine	sheep	25.6	9.4									
Ovdat	A-3141	1143	171	L Roman/ E Byzantine	shg		9.3									

Ovdat	A-3141	1143	171	L Roman/ E Byzantine	sheep	29.4	10.3									
Astragalus						GLl	Bd	Dl								
Nizzana	G4/17	103	1135/2	Early Islamic	gazelle	24.4	15.6	14.3								
Nizzana	G4/17	105	1236/1	Early Islamic	gazelle	26.8	16.1	15.2								
Nizzana	G4/17	105	1236/1	Early Islamic	gazelle	27.3	16.8	15.4								
Nizzana	G4/17	103	1135/2	Early Islamic	gazelle	27.9	1.2	16.2								
Nizzana	G4/17	119	1398/7	Byzantine	goat	29.8	19.4	15.9								
Nizzana	G4/17	104	1210/2	Early Islamic	goat		20.5	17.4								
Nizzana	G4/17	103	1177/4	Early Islamic	pig	43.8										
Nizzana	G4/17	102	1082/1	Early Islamic	pig	49.7										
Nizzana	G4/17	101	1005/1	Early Islamic	pig	51.2										
Haluza	G10/15	4/07d	72	Byzantine	sheep	30.5	19.3	17								
Haluza	G10/15	1/01e	100	Byzantine	sheep	31.9	20.8	18								
Haluza	G-10/15	01/07	42	Byzantine	sheep	33.7	21.4	18.1								
Nizzana	G4/17	309	3075/1	Byzantine	sheep	35.7		19.1								
Nizzana	G4/17	102	1098/2	Early Islamic	sheep	31.1	19.3	16.7								
SH	G87/15	156	1574	Early Islamic	sheep	33.7	21.8	18.3								

**SH**	**G87/ 15**	**904**	**9070**		**sheep**	**35**	**21.4**	**19.2**								
**SH**	**G87/ 15**	**909**	**9053**		**shg**	**31.8**	**21.2**	**17.9**								
**Ovdat**	**A-3141**	**1142**	**149**	**L Roman/E Byzantine**	**?antelop e**	**38.2**	**25**	**20.7**								
**Ovdat**	**A-3141**	**704**	**84**	**L Roman/E Byzantine**	**camel**	**75.8**	**52.6**	**42.7**								
**Ovdat**	**A-3141**	**6362**	**706**	**L Roman/E Byzantine**	**donkey**	**57.3**	**47.7**									
**Ovdat**	**A-3141**	**1122**	**126**	**L Roman/E Byzantine**	**goat**	**27.2**	**18.1**	**14.7**								
**Ovdat**	**A-3141**	**1122**	**126**	**L Roman/E Byzantine**	**goat**	**29.7**	**18.2**	**15.4**								
**Ovdat**	**A-3141**	**3307**	**31688**	**L Roman/E Byzantine**	**goat**	**31.8**	**20.2**	**17.9**								
**Ovdat**	**A-3141**	**1102**	**46**	**L Roman/E Byzantine**	**goat**	**36.5**	**23.7**	**21.3**								
**Ovdat**	**A-3141**	**702**	**63**	**L Roman/E Byzantine**	**goat**	**36**	**22.6**	**18.7**								
**Ovdat**	**A-3141**	**1122**	**126**	**L Roman/E Byzantine**	**sheep**		**23.4**									
**Ovdat**	**A-3141**	**1122**	**126**	**L Roman/E Byzantine**	**sheep**		**24.1**									
**Ovdat**	**A-3141**	**1122**	**126**	**L Roman/E Byzantine**	**sheep**	**30.7**	**20.5**	**17**								
**Ovdat**	**A-3141**	**1143**	**171**	**L Roman/E Byzantine**	**sheep**	**30.9**	**19.9**	**17.5**								
**Ovdat**	**A-3141**	**1122**	**126**	**L Roman/E Byzantine**	**sheep**	**31.5**	**20.4**	**17.4**								
**Ovdat**	**A-3141**	**3307**	**31688**	**L Roman/E Byzantine**	**sheep**	**32.4**	**20.3**	**18.2**								
**Ovdat**	**A-3141**	**1122**	**126**	**L Roman/E Byzantine**	**sheep**	**32.6**	**21**	**18.1**								

Ovdat	A-3141		281	L Roman/E Byzantine	sheep	32.7	22.1	18.5								
Ovdat	A-3141	701	14	L Roman/E Byzantine	sheep	33.2	21.3	18.4								
Ovdat	A-3141	1143	171	L Roman/E Byzantine	sheep	33.5	21	17.1								
Ovdat	A-3141	1132	137	L Roman/E Byzantine	sheep	34.2	22.6	19.2								
Ovdat	A-3141	171	1143	L Roman/E Byzantine	sheep	34.6	22.6	18.1								
Ovdat	A-3141	1132	137	L Roman/E Byzantine	sheep	36.1	24	19.7								
Ovdat	A-3141	1132	137	L Roman/E Byzantine	sheep	36.3	24	19.1								
Ovdat	A-3141	1142	161	L Roman/E Byzantine	shg	33.5	23.1	19.4								
Ovdat	A-3141	1202	43	L Roman/E Byzantine	shg	35.6										
Calcaneus						GL										
Nizzana	G4/17	103	1135/2	Early Islamic	goat	58.7										
Nizzana	G4/17	103	1135/2	Early Islamic	goat	60.3										
Nizzana	G4/17	103	1177/4	Early Islamic	goat	60.8										
SH	G87/15	508	5044		goat	56.9										
SH	G87/15	508	5044		goat	60.2										
Haluza	G-10/15	1/07a	41	Byzantine	sheep	68.9										
Haluza	G-10/15	1/06a	33	Byzantine	sheep	62.7										

**Ovdat**	**A-3141**	**1122**	**126**	**L Roman/E Byzantine**	**goat**	**55.6**										
**Ovdat**	**A-3141**	**1122**	**116**	**L Roman/E Byzantine**	**goat**	**62.6**										
**Ovdat**	**A-3141**	**"mezaveh"**		**L Roman/E Byzantine**	**goat**	**56.1**										
**Ovdat**	**A-3141**	**1122**	**126**	**L Roman/E Byzantine**	**sheep**	**59.2**										
**Ovdat**	**A-3141**	**1122**	**116**	**L Roman/E Byzantine**	**sheep**	**59.6**										
**Ovdat**	**A-3141**	**4601**	**262**	**L Roman/E Byzantine**	**sheep**	**63.6**										
**Ovdat**	**A-3141**	**1202**	**40**	**L Roman/E Byzantine**	**sheep**	**71**										
**Ovdat**	**A-3141**	**1202**	**40**	**L Roman/E Byzantine**	**sheep**	**72.1**										
**Ovdat**	**A-3141**	**1102**	**145**	**L Roman/E Byzantine**	**sheep**	**72.7**										
**Carpometacarpus**						**GL**										
**Haluza**	**G10/15**	**7/01d**	**85**	**Byzantine**	**chicken**	**34.8**										
**Nizzana**	**G4/17**	**103**	**1177/4**	**Early Islamic**	**chicken**	**35.5**										
**Nizzana**	**G4/17**	**101**	**1048/3**	**Early Islamic**	**pigeon**	**30**										
**Ovdat**	**A-3141**	**"mezaveh"**		**L Roman/E Byzantine**	**chicken**	**39.9**										
**Coracoid**						**GLl**										
**Haluza**	**G10/15**	**1/09b**	**53**	**Byzantine**	**chicken**	**50.8**										
**SH**	**G87/15**	**169**	**1676**	**Byzantine**	**chicken**	**53.4**										
**Nizzana**	**G4/17**	**103**	**1135/2**	**Early Islamic**	**chicken**	**53.9**										

**Nizzana**	**G4/17**	**105**	**1236/1**	**Early Islamic**	**chicken**	**48.3**										
**Nizzana**	**G4/17**	**119**	**1398/12**	**Byzantine**	**pigeon**	**28.7**										
**dp4**						**L**										
**Haluza**	**G10/15**	**6/01a**	**74**	**Byzantine**	**pig**	**18.6**										
**SH**	**G87/15**	**550**	**?**		**pig**	**19.1**										
**Haluza**	**G-10/15**	**4/03c**	**27**	**Byzantine**	**sheep**	**19.2**										
**Femur**						**GL**	**Bd**									
**Haluza**	**G-10/15**	**1/06a**	**33**	**Byzantine**	**chicken**	**76.8**	**15.2**									
**Haluza**	**G10/15**	**7/01b**	**83**	**Byzantine**	**chicken**	**78.6**	**15.8**									
**Nizzana**	**G4/17**	**107**	**1295**	**Early Islamic**	**chicken**		**13.9**									
**Nizzana**	**G4/17**	**105**	**1236/1**	**Early Islamic**	**chicken**		**14.2**									
**Nizzana**	**G4/17**	**103**	**1135/2**	**Early Islamic**	**chicken**	**82.7**	**16.3**									
**SH**	**G87/15**	**706**	**7011**		**chicken**	**70.6**	**14.2**									
**Nizzana**	**G4/17**	**103**	**1156/3**	**Early Islamic**	**gazelle**		**29.8**									
**SH**	**G87/15**	**409**	**4061**		**shg**		**43.3**									
**Ovdat**	**A-3141**	**701-702**	**room 3**	**L Roman/E Byzantine**	**chicken**	**72.2**	**13.7**									
**Ovdat**	**A-3141**	**1142**	**149**	**L Roman/E Byzantine**	**chicken**	**75.7**										
**Ovdat**	**A-3141**	**1122**	**116**	**L Roman/E Byzantine**	**shg**		**38.7**									

**Ovdat**	**A-3141**	**1122**	**116**	**L Roman/E Byzantine**	**shg**		**40.3**									
**Ovdat**	**A-3141**	**"mezaveh"**		**L Roman/E Byzantine**	**shg**		**43.9**									
**Humerus**						**GL**	**Bp**	**Bd**	**BT**	**HTC**						
**Nizzana**	**G4/17**	**103**	**1177/4**	**Early Islamic**	**cat**			**18**								
**Haluza**	**G10/15**	**4/07d**	**72**	**Byzantine**	**chicken**			**12**								
**Haluza**	**G-10/15**	**1/06b**	**34**	**Byzantine**	**chicken**			**14.6**								
**Haluza**	**G-10/15**	**7/01a**	**82**	**Byzantine**	**chicken**			**16**								
**Nizzana**	**G4/17**	**507**	**5087**	**Byzantine**	**chicken**		**19.2**									
**Nizzana**	**G4/17**	**103**	**1177/4**	**Early Islamic**	**galliforme**			**13.5**								
**Nizzana**	**G4/17**	**121**	**1484/2**	**Byzantine**	**gazelle**				**23**	**13.9**						
**Nizzana**	**G4/17**	**103**	**1177/4**	**Early Islamic**	**gazelle**				**22.7**	**12.8**						
**Nizzana**	**G4/17**	**103**	**1156/3**	**Early Islamic**	**gazelle**				**24.4**	**12.8**						
**Nizzana**	**G4/17**	**103**	**1135/2**	**Early Islamic**	**gazelle**				**24.5**	**13.1**						
**Nizzana**	**G4/17**	**103**	**1177/4**	**Early Islamic**	**gazelle**					**12.2**						
**Nizzana**	**G4/17**	**505**	**5074**	**Byzantine**	**goat**				**31.4**	**14.2**						
**SH**	**G4/16**	**163**	**1637**	**Byzantine**	**goat**					**16.8**						
**Nizzana**	**G4/17**	**103**	**1156/3**	**Early Islamic**	**goat**				**32.4**	**14.9**						

**Nizzana**	**G4/17**	**123**	**1435/1**	**Byzantine**	**hare**	**82.4**		**9.5**		**5.3**						
**SH**	**G87/15**	**700**	**7000**		**hare**			**8.8**		**5.1**						
**Haluza**	**G-10/15**	**4/03a**	**25**	**Byzantine**	**pig**					**22**						
**Nizzana**	**G4/17**	**103**	**1177/4**	**Early Islamic**	**pig**				**29.9**	**21.6**						
**Nizzana**	**G4/17**	**103**	**1135/2**	**Early Islamic**	**pigeon**	**44.1**		**10.8**								
**Haluza**	**G-10/15**	**1/07a**	**41**	**Byzantine**	**sheep**				**34.6**	**18.1**						
**Nizzana**	**G4/17**	**115**	**1344/1**	**Byzantine**	**sheep**				**30.5**	**15.7**						
**Nizzana**	**G4/17**	**103**	**1156/3**	**Early Islamic**	**sheep**				**31.4**	**14.7**						
**Haluza**	**G-10/15**	**4/02b**	**17**	**Byzantine**	**shg**				**33.1**	**16.4**						
**Ovdat**	**A-3141**	**701**	**27**	**L Roman/E Byzantine**	**chicken**			**13.1**								
**Ovdat**	**A-3141**	**1122**	**116**	**L Roman/E Byzantine**	**chicken**	**65.1**		**13.4**								
**Ovdat**	**A-3141**	**1122**	**116**	**L Roman/E Byzantine**	**chicken**	**69.8**		**14.7**								
**Ovdat**	**A-3141**	**"mezaveh"**		**L Roman/E Byzantine**	**chicken**	**71.5**		**14.5**								
**Ovdat**	**A-3141**	**"mezaveh"**		**L Roman/E Byzantine**	**chicken**	**74.5**		**14.7**								
**Ovdat**	**A-3141**	**1122**	**116**	**L Roman/E Byzantine**	**goat**				**30.1**	**15.1**						
**Ovdat**	**A-3141**	**1122**	**126**	**L Roman/E Byzantine**	**pigeon**	**43.6**		**9.9**								
**Ovdat**	**A-3141**	**1122**	**126**	**L Roman/E Byzantine**	**sheep**				**29.1**	**14**						

**Ovdat**	**A-3141**	**1101**	**7**	**L Roman/E Byzantine**	**sheep**				**29.9**							
**Ovdat**	**A-3141**	**1122**	**116**	**L Roman/E Byzantine**	**sheep**				**30.1**	**14.8**						
**Ovdat**	**A-3141**	**1122**	**116**	**L Roman/E Byzantine**	**sheep**				**30.5**	**15.2**						
**Ovdat**	**A-3141**	**"mezaveh"**		**L Roman/E Byzantine**	**sheep**				**30.6**	**16.4**						
**Ovdat**	**A-3141**	**1101**	**7**	**L Roman/E Byzantine**	**sheep**				**31.6**	**15.6**						
**Ovdat**	**A-3141**	**704**	**87**	**L Roman/E Byzantine**	**sheep**				**31.7**	**16.4**						
**Ovdat**	**A-3141**	**1102**	**41**	**L Roman/E Byzantine**	**sheep**				**31**	**15.8**						
**Ovdat**	**A-3141**	**1122**	**126**	**L Roman/E Byzantine**	**sheep**				**32.3**	**16.6**						
**Ovdat**	**A-3141**	**1302**	**290**	**L Roman/E Byzantine**	**sheep**				**33.3**	**17**						
**Ovdat**	**A-3141**	**1142**	**161**	**L Roman/E Byzantine**	**sheep**				**34.2**	**18**						
**Ovdat**	**A-3141**	**1142**	**161**	**L Roman/E Byzantine**	**sheep**				**34.3**	**18.5**						
**Ovdat**	**A-3141**	**1122**	**126**	**L Roman/E Byzantine**	**sheep**				**35.1**	**17**						
**Ovdat**	**A-3141**	**1302**	**290**	**L Roman/E Byzantine**	**sheep**				**35.2**	**17.7**						
**Ovdat**	**A-3141**	**702**	**63**	**L Roman/E Byzantine**	**sheep**				**36.3**	**18.5**						
**Ovdat**	**A-3141**	**1122**	**126**	**L Roman/E Byzantine**	**sheep**					**14.6**						
**Ovdat**	**A-3141**	**1202**	**40**	**L Roman/E Byzantine**	**sheep**					**17**						
**Ovdat**	**A-3141**	**701-702**	**room 3**	**L Roman/E Byzantine**	**shg**				**30.4**	**15.1**						

**Ovdat**	**A-<br>3141**	**1122**	**116**	**L Roman/E<br>Byzantine**	**shg**				**31.7**	**15.3**						
**Ovdat**	**A-<br>3141**	**1132**	**137**	**L Roman/E<br>Byzantine**	**shg**				**32.6**							
**Ovdat**	**A-<br>3141**	**1202**	**40**	**L Roman/E<br>Byzantine**	**shg**				**33.4**							
**Ovdat**	**A-<br>3141**	**1143**	**171**	**L Roman/E<br>Byzantine**	**shg**				**35.2**							
**Ovdat**	**A-<br>3141**	**1122**	**109**	**L Roman/E<br>Byzantine**	**shg**					**15.4**						
**Ovdat**	**A-<br>3141**	**1102**	**41**	**L Roman/E<br>Byzantine**	**shg**					**15.8**						
**Ovdat**	**A-<br>3141**	**704**	**84**	**L Roman/E<br>Byzantine**	**shg**					**18**						
**Metacarpus**						**GL**	**Bd**	**BFd**	**DE<br>M**	**DV<br>M**	**WC<br>M**	**DEL**	**DV<br>L**	**WC<br>L**	**Dd**	**SD**
**Haluz<br>a**	**G10/<br>15**	**1/09b**	**53**	**Byzantine**	**antelope**		**34.5**	**30.9**	**14.4**		**13.7**					
**Haluz<br>a**	**G10/<br>15**	**1/09b**	**53**	**Byzantine**	**goat**			**25.6**	**9.9**	**17**	**11.9**					
**Nizza<br>na**	**G4/1<br>7**	**113**	**1312/<br>1**	**Byzantine**	**goat**		**27.2**	**27.8**	**10.3**	**17.7**	**12.7**	**10.5**		**13**		
**Nizza<br>na**	**G4/1<br>7**	**110**	**1326/<br>1**	**Byzantine**	**goat**		**27.9**									
**Nizza<br>na**	**G4/1<br>7**	**202**	**2004/<br>2**	**Early Islamic**	**goat**	**129.6**	**26.1**	**26.8**	**11.9**	**17.7**	**12.8**	**11.4**	**17.2**	**12.6**		
**SH**	**G87/<br>15**	**413**	**4083**		**goat**							**9.8**	**18**	**12.8**		
**Nizza<br>na**	**G4/1<br>7**	**112**	**1329/<br>1**	**Byzantine**	**pig<br>(MC3)**		**15.1**									
**Nizza<br>na**	**G4/1<br>7**	**107**	**1295**	**Early Islamic**	**pig<br>(MC3)**		**12.6**									
**SH**	**G87/<br>15**	**904**	**9070**		**pig<br>(MC3)**		**14.9**									

**Haluz<br>a**	**G-<br>10/15**	**4/05a**	**45**	**Byzantine**	**sheep**			**30.4**	**13**	**19.3**	**14**	**13.9**		**14.3**		
**Haluz<br>a**	**G-<br>10/15**	**1/05c**	**35**	**Byzantine**	**sheep**							**14.6**	**20.4**	**14.1**		
**Haluz<br>a**	**G-<br>10/15**	**1/06b**	**34**	**Byzantine**	**sheep**				**12.5**	**17.4**	**13**					
**Nizza<br>na**	**G4/1<br>7**	**103**	**1177/<br>4**	**Early Islamic**	**sheep**				**12.9**	**19.2**	**13.3**					
**SH**	**G87/<br>15**	**909**	**9053**		**sheep**				**12.2**		**13**					
**Nizza<br>na**	**G4/1<br>7**	**114**	**1339/<br>1**	**Byzantine**	**shg**		**28.8**									
**Ovdat**	**A-<br>3141**	**1143**	**171**	**L Roman/E<br>Byzantine**	**goat**							**12**	**21.3**	**16.1**		
**Ovdat**	**A-<br>3141**	**1143**	**171**	**L Roman/E<br>Byzantine**	**goat**				**11.5**	**21**	**15.9**					
**Ovdat**	**A-<br>3141**	**1142**	**161**	**L Roman/E<br>Byzantine**	**goat**			**30.4**	**11.3**	**20.1**	**14.4**	**12.6**	**19.6**	**13.7**		
**Ovdat**	**A-<br>3141**	**704**	**84**	**L Roman/E<br>Byzantine**	**goat**			**34**	**12.1**	**21.7**	**16.1**	**11.8**	**21.7**	**15.4**		
**Ovdat**	**A-<br>3141**	**3307**	**31688**	**L Roman/E<br>Byzantine**	**goat**			**26.4**	**9.1**	**16.7**	**12.5**	**9.3**	**16.5**	**12.2**		
**Ovdat**	**A-<br>3141**	**1121**	**100**	**L Roman/E<br>Byzantine**	**goat**			**29.5**	**13.5**	**19.1**	**13.5**	**12.5**	**18.4**	**13.4**		
**Ovdat**	**A-<br>3141**	**1102**	**29**	**L Roman/E<br>Byzantine**	**sheep**			**29.5**	**12.7**	**19.6**	**13.7**	**11.8**	**18.6**	**12.4**		
**Ovdat**	**A-<br>3141**	**4042**	**638**	**L Roman/E<br>Byzantine**	**sheep**			**29**	**12.5**	**18.7**	**13.7**	**12**	**18.1**	**12.5**		
**Ovdat**	**A-<br>3141**	**1122**	**126**	**L Roman/E<br>Byzantine**	**sheep**			**31.6**	**13.6**	**19.1**	**14.2**	**14**	**19.8**	**14.8**		
**Ovdat**	**A-<br>3141**	**1202**	**43**	**L Roman/E<br>Byzantine**	**sheep**			**24.7**	**12.3**	**16.9**	**11.7**	**11.6**	**16.4**	**10.9**		
**Ovdat**	**A-<br>3141**	**"mezave<br>h"**		**L Roman/E<br>Byzantine**	**sheep**			**26.5**	**11.5**	**17.2**	**12.4**	**11.3**	**16.6**	**12.1**		

**Metatarsus**						**GL**	**Bd**	**BFd**	**DEM**	**DVM**	**WCM**	**DEL**	**DVL**	**WCL**	**Dd**	**SD**
Nizzana	G4/17	103	1156/3	Early Islamic	equid		36.6	37							27.1	26.1
Nizzana	G4/17	405	4039/1	Byzantine/Early Islamic	gazelle		19.5									
Nizzana	G4/17	103	1218/5	Early Islamic	gazelle		18.6	19.8	10.3		9	10.8		9.1		
Nizzana	G4/17	105	1236/1	Early Islamic	gazelle		19.2	19.7	10.8		9.1					
Nizzana	G4/17	103	1177/4	Early Islamic	gazelle	174	19.6	20.7	10.8		9.5	10.2		9.3		10.4
Haluza	G-10/15	1/04a	20	Byzantine	goat		31.9	31								
Haluza	G-10/15	1/06a	33	Byzantine	goat			24								
Haluza	G-10/15	4/04b	38	Byzantine	goat			26	9.7		12.4	9.1	16.6	12		
Haluza	G10/15	1/09b	53	Byzantine	goat				9.4	17.1	12.7					
Nizzana	G4/17	122	1426/1	Byzantine	goat		25.1	24.8	9.6	11.8	11.8	9.6	16.3	11		
Nizzana	G4/17	103	1177/4	Early Islamic	goat							10.4	17.7	13.2		
Haluza	G-10/15	7/05d	99	Byzantine	sheep		25.5	25.2	12		12.1	11.8	16.8	11.1		
Haluza	G10/15	1/09b	53	Byzantine	sheep		26.6									
Haluza	G10/15	1/01e	100	Byzantine	sheep			24.9	11.3	16.6	12.2					
Haluza	G10/15	6/01b	75	Byzantine	sheep			16.4	11.3	17.3	12.8	10.5	16.3	11.6		

**Haluza**	**G-10/15**	**1/06a**	**33**	**Byzantine**	**sheep**			**26.9**	**11.4**	**16.3**	**12.5**	**12.2**	**17**	**12.9**		
**Haluza**	**G-10/15**	**1/05b**	**32**	**Byzantine**	**sheep**			**30.6**	**13**	**20.3**	**14.8**	**12.9**	**19.6**	**13.2**		
**Nizzana**	**G4/17**	**121**	**1469/1**	**Byzantine**	**sheep**			**28.6**				**10.3**	**16.3**	**11.9**		
**Nizzana**	**G4/17**	**103**	**1135/2**	**Early Islamic**	**sheep**		**26.9**	**27.4**	**12.8**	**18.5**	**13**	**11.8**	**17.3**	**12**		
**SH**	**G87/15**	**550**	**5532**		**sheep**				**10.5**		**12.7**					
**Haluza**	**G10/15**	**1/09b**	**53**	**Byzantine**	**shg**		**26**									
**Nizzana**	**G4/17**	**121**	**1469/1**	**Byzantine**	**shg**		**26.1**									
**Ovdat**	**A-3141**	**1142**	**1900/5**	**L Roman/E Byzantine**	**?antelope**							**14.3**	**21**	**15.5**		
**Ovdat**	**A-3141**	**1122**	**1900/4**	**L Roman/E Byzantine**	**sheep**				**10.8**	**15.8**	**11.2**					
**Ovdat**	**A-3141**	**1202**	**1900/2**	**L Roman/E Byzantine**	**sheep**				**11.4**	**16.9**	**11.6**					
**Ovdat**	**A-3141**	**1142**	**1900/6**	**L Roman/E Byzantine**	**shg**							**10.3**	**16.9**	**11.5**		
**Ovdat**	**A-3141**	**1122**	**1900/4**	**L Roman/E Byzantine**	**?hartebeest**			**29.1**	**13.9**	**21.1**	**13.9**	**13**	**19.8**	**12.3**		
**Ovdat**	**A-3141**	**1102**	**1900/2**	**L Roman/E Byzantine**	**cattle**				**24.6**	**34.1**	**29**					
**Ovdat**	**A-3141**	**1122**	**1900/5**	**L Roman/E Byzantine**	**goat**		**25.8**	**25.8**	**9.9**	**17.5**	**12.3**	**10.3**	**17.3**	**11.5**		
**Ovdat**	**A-3141**	**1122**	**1900/5**	**L Roman/E Byzantine**	**goat**			**28.2**	**10.4**	**18.3**	**13**	**11.1**	**18**	**12.7**		
**Ovdat**	**A-3141**	**"mezaveh"**		**L Roman/E Byzantine**	**goat**			**24.4**	**9.5**	**6.8**	**11.2**	**10.3**	**16.7**	**10.7**		
**Ovdat**	**A-3141**	**1122**	**1900/4**	**L Roman/E Byzantine**	**goat**	**120.8**		**24.8**	**9.2**	**16.6**	**11.4**	**9.4**	**16.3**	**10.9**		

**Ovdat**	**A-3141**	**701-702**	**room 3**	**L Roman/E Byzantine**	**sheep**			**24.7**	**11.3**	**17.2**	**12**	**10.3**	**16.1**	**10.7**		
**Ovdat**	**A-3141**	**1122**	**1900/5**	**L Roman/E Byzantine**	**sheep**			**25.5**	**11**	**16.4**	**11.8**	**10.9**	**15.6**	**10.6**		
**Ovdat**	**A-3141**	**6362**	**1901/12**	**L Roman/E Byzantine**	**sheep**			**26.8**	**12**		**12.6**	**11.6**	**18.2**	**11.4**		
**Ovdat**	**A-3141**	**701-702**	**room 3**	**L Roman/E Byzantine**	**sheep**			**26.8**	**12.1**	**17.4**	**12.7**	**11.6**	**16.8**	**12.5**		
**Ovdat**	**A-3141**	**1101**	**1900/1**	**L Roman/E Byzantine**	**sheep**			**27.4**	**11**	**17.4**	**12**					
**Ovdat**	**A-3141**	**6362**	**1901/12**	**L Roman/E Byzantine**	**sheep**			**28.1**	**11.8**	**18.1**	**12.7**	**11.2**	**17.2**	**11.3**		
**Ovdat**	**A-3141**	**1132**	**1900/5**	**L Roman/E Byzantine**	**sheep**			**28.1**	**12.4**	**19.5**	**13.1**	**12.2**	**18.8**	**11.8**		
**Ovdat**	**A-3141**	**704**	**1900/3**	**L Roman/E Byzantine**	**sheep**			**28.5**	**13.5**	**19.3**	**12.8**	**12.7**	**18.5**	**11.7**		
**Ovdat**	**A-3141**	**1142**	**1900/6**	**L Roman/E Byzantine**	**sheep**			**29.1**	**12.9**	**19.8**	**13.7**	**12.6**	**19.1**	**12.6**		
**Ovdat**	**A-3141**	**1202**	**1900/2**	**L Roman/E Byzantine**	**sheep**			**23.7**	**10.8**	**16.6**	**11.5**	**10.2**	**15.8**	**10.7**		
**Phalanx 1**						**GL/GLpe**	**Bp**	**Bd**	**SD**							
**Nizzana**	**G4/17**	**103**	**1218/5**	**Early Islamic**	**cattle**			**29.8**	**27.9**							
**Nizzana**	**G4/17**	**115**	**1387/1**	**Byzantine**	**chicken**	**17.8**										
**Nizzana**	**G4/17**	**103**	**1135/2**	**Early Islamic**	**chicken**	**17.9**										
**SH**	**G87/15**	**401**	**4026**		**dog**	**27.4**	**9.6**	**8.1**	**5.8**							
**Nizzana**	**G4/17**	**103**	**1218/5**	**Early Islamic**	**gazelle**	**40.6**	**9.2**	**8.8**	**6.9**							
**Nizzana**	**G4/17**	**101**	**1035/2**	**Early Islamic**	**gazelle**	**43.8**	**9.9**	**9.4**	**7.8**							

Haluza	G10/15	7/01c	84	Byzantine	goat	40.1	13.5	12.1								
Haluza	G-10/15	4/05c	47	Byzantine	goat	40.8	13.1	12.7								
Haluza	G-10/15	1/04c	31	Byzantine	goat	37.2	13.1	12.7								
Haluza	G-10/15	1/02d	30	Byzantine	goat	37	13.4	12.7								
Haluza	G-10/15	1/06b	34	Byzantine	goat	37	13.6	13.3								
Haluza	G-10/15	1/03d	36	Byzantine	goat	40.8	13.4	13.1	10.5							
Nizzana	G4/17	112	1329/1	Byzantine	goat			14.7								
Nizzana	G4/17	131	1593/1	Byzantine	goat		14.5									
Nizzana	G4/17	115	1387/1	Byzantine	goat	35.8	12.9	13.1	11.3							
Nizzana	G4/17	318	3138	Byzantine	goat	36.6	13.8	14	12							
Nizzana	G4/17	119	1398/11	Byzantine	goat	37.5	14.6	14.2	12.2							
Nizzana	G4/17	113	1312/1	Byzantine	goat	41.1	13.6	12	10.5							
Nizzana	G4/17	503	5048	Byzantine/Early Islamic	goat	43.4	14	13	11.2							
Nizzana	G4/17	104	1210/2	Early Islamic	goat			13.4								
Nizzana	G4/17	108	1278/1	Early Islamic	goat	36.3	12.9	11.6	10.4							
SH	G87/15	409	4059		goat			14.3								
SH	G87/15	909	9053		goat	38.3	14.8	13.1	10.3							

**SH**	**G87/15**	**408**	**4046**		**goat**	**38.4**	**14.5**	**14.6**	**12**							
**SH**	**G87/15**	**410**	**4071**		**goat**	**39.3**		**11.7**								
**Haluza**	**G10/15**	**6/01d**	**77**	**Byzantine**	**pig**	**34.8**	**14.3**	**14.4**								
**Nizzana**	**G4/17**	**127**	**1586/1**	**Byzantine**	**pig**		**20.7**									
**Nizzana**	**G4/17**	**103**	**1121/1**	**Early Islamic**	**pig**			**19.1**								
**SH**	**G87/15**	**550**	**?**		**pig**		**13.3**									
**SH**	**G87/15**	**508**	**5044**		**pig**		**15.8**	**15.9**								
**Nizzana**	**G4/17**	**307**	**3052/1**	**Byzantine**	**sheep**		**13.6**	**13.9**	**11.4**							
**Haluza**	**G-10/15**	**1/01d**	**23**	**Byzantine**	**sheep**		**12.5**	**12.2**								
**Nizzana**	**G4/17**	**121**	**1484/2**	**Byzantine**	**sheep**	**43.9**	**16.6**	**15**	**13.2**							
**Nizzana**	**G4/17**	**103**	**1177/4**	**Early Islamic**	**sheep**			**13.9**								
**Nizzana**	**G4/17**	**103**	**1135/2**	**Early Islamic**	**sheep**		**13.3**	**13.1**								
**Nizzana**	**G4/17**	**105**	**1236/1**	**Early Islamic**	**sheep**	**38.2**	**13.7**	**12.9**	**9.9**							
**SH**	**G4/16**	**156**	**1591**	**Early Islamic**	**sheep**	**41.2**	**14**	**12.8**	**11**							
**SH**	**G87/15**	**409**	**4061**		**sheep**			**13.3**								
**Haluza**	**G-10/15**	**1/04b**	**21**	**Byzantine**	**shg**		**13.1**									

**Haluza**	**G-10/15**	**4/05a**	**45**	**Byzantine**	**shg**		**13.5**									
**Haluza**	**G-10/15**	**1/06b**	**34**	**Byzantine**	**shg**	**37**	**13.9**	**12.8**								
**Nizzana**	**G4/17**	**113**	**1312/1**	**Byzantine**	**shg**			**10.2**								
**Nizzana**	**G4/17**	**103**	**1135/2**	**Early Islamic**	**shg**		**13.6**									
**SH**	**G87/15**	**650**	**6504**		**shg**		**13.7**									
**SH**	**G87/15**	**909**	**9053**		**shg**		**16.9**									
**SH**	**G87/15**	**502**	**5009**		**shg**	**37.2**	**13.3**	**13.6**	**11.7**							
**Ovdat**	**A-3141**	**"mezaveh"**		**L Roman/E Byzantine**	**shg**	**37.4**	**12.9**	**13.1**	**10.3**							
**Ovdat**	**A-3141**	**1142**	**149**	**L Roman/E Byzantine**	**?antelope**	**43.4**	**16**	**15.3**	**13.1**							
**Ovdat**	**A-3141**	**1122**	**116**	**L Roman/E Byzantine**	**camel**	**105.8**										
**Ovdat**	**A-3141**	**1923**	**260**	**L Roman/E Byzantine**	**camel**	**108.5**	**48.2**									
**Ovdat**	**A-3141**	**1122**	**126**	**L Roman/E Byzantine**	**camel**	**92.4**	**39.1**	**34.8**	**20.5**							
**Ovdat**	**A-3141**	**1101**	**7**	**L Roman/E Byzantine**	**cattle**			**26.4**								
**Ovdat**	**A-3141**	**702**	**63**	**L Roman/E Byzantine**	**cattle**		**26.4**									
**Ovdat**	**A-3141**	**1102**	**59**	**L Roman/E Byzantine**	**donkey**	**74.1**	**39.3**	**34.6**	**24.7**							
**Ovdat**	**A-3141**	**1122**	**126**	**L Roman/E Byzantine**	**goat**			**13.1**								
**Ovdat**	**A-3141**	**1122**	**126**	**L Roman/E Byzantine**	**goat**		**12**	**12.8**								

**Ovdat**	**A-3141**	**1122**	**126**	**L Roman/E Byzantine**	**goat**	**36.6**	**12.4**	**12.5**	**9.9**							
**Ovdat**	**A-3141**	**704**	**87**	**L Roman/E Byzantine**	**goat**	**37.4**	**12.5**	**12.6**	**11.1**							
**Ovdat**	**A-3141**	**1122**	**116**	**L Roman/E Byzantine**	**goat**	**38.6**	**13.9**	**13.6**	**12.2**							
**Ovdat**	**A-3141**	**1122**	**126**	**L Roman/E Byzantine**	**goat**	**39.8**	**12.7**	**13.2**	**11.3**							
**Ovdat**	**A-3141**	**702**	**53**	**L Roman/E Byzantine**	**goat**	**40.2**	**12.2**	**11.7**	**9.7**							
**Ovdat**	**A-3141**	**1101**	**7**	**L Roman/E Byzantine**	**goat**	**40.7**	**14.1**	**13.5**	**11.5**							
**Ovdat**	**A-3141**	**702**	**63**	**L Roman/E Byzantine**	**goat**	**41.2**	**13.3**	**11.9**	**10.2**							
**Ovdat**	**A-3141**	**701-702**	**room 3**	**L Roman/E Byzantine**	**goat**	**41**	**14**	**13.6**	**11**							
**Ovdat**	**A-3141**	**4601**	**262**	**L Roman/E Byzantine**	**goat**	**42.4**	**14**	**12.8**	**11.4**							
**Ovdat**	**A-3141**	**1122**	**116**	**L Roman/E Byzantine**	**goat**	**42**	**13.9**	**13**	**11.4**							
**Ovdat**	**A-3141**	**6362**	**7121**	**L Roman/E Byzantine**	**goat**	**43.5**	**13.8**	**13.1**	**10.5**							
**Ovdat**	**A-3141**	**1122**	**116**	**L Roman/E Byzantine**	**goat**	**47.2**	**16.2**	**15.3**	**13.6**							
**Ovdat**	**A-3141**	**1101**	**7**	**L Roman/E Byzantine**	**sheep**			**13.5**								
**Ovdat**	**A-3141**	**1132**	**137**	**L Roman/E Byzantine**	**sheep**			**13.6**								
**Ovdat**	**A-3141**	**1102**	**145**	**L Roman/E Byzantine**	**sheep**	**37.2**	**11.7**	**10.7**	**9.9**							
**Ovdat**	**A-3141**	**3307**	**31688**	**L Roman/E Byzantine**	**sheep**	**38.2**	**13.7**	**12.2**	**11**							
**Ovdat**	**A-3141**	**1143**	**171**	**L Roman/E Byzantine**	**sheep**	**39**	**13.2**	**12.1**	**10**							

**Ovdat**	**A-3141**	**703**	**74**	**L Roman/E Byzantine**	**sheep**	**40.4**	**12.5**	**11.5**	**9.8**							
**Ovdat**	**A-3141**	**1122**	**116**	**L Roman/E Byzantine**	**sheep**	**40.9**	**14.2**	**14.1**	**12**							
**Ovdat**	**A-3141**	**3307**	**31688**	**L Roman/E Byzantine**	**sheep**	**40.9**	**14.6**	**13.8**	**12.1**							
**Ovdat**	**A-3141**	**1202**	**43**	**L Roman/E Byzantine**	**sheep**	**41**	**14.8**	**12.8**	**11.5**							
**Ovdat**	**A-3141**	**6362**	**7121**	**L Roman/E Byzantine**	**sheep**	**42.7**	**13.4**	**12.1**	**10.4**							
**Ovdat**	**A-3141**	**"mezaveh"**		**L Roman/E Byzantine**	**sheep**	**42.9**	**14.2**	**14.1**	**12**							
**Ovdat**	**A-3141**	**1101**	**7**	**L Roman/E Byzantine**	**shg**		**14**									
**Ovdat**	**A-3141**	**1132**	**137**	**L Roman/E Byzantine**	**shg**		**14.6**									
**Ovdat**	**A-3141**	**1102**	**41**	**L Roman/E Byzantine**	**shg**		**16**									
**Ovdat**	**A-3141**	**1122**	**126**	**L Roman/E Byzantine**	**shg**	**33.3**	**11.6**	**11.8**	**9.6**							
**Ovdat**	**A-3141**	**701**	**20**	**L Roman/E Byzantine**	**shg**	**39.5**	**14**	**14.3**	**11.6**							
**Ovdat**	**A-3141**	**1102**	**60**	**L Roman/E Byzantine**	**shg**	**41**	**14.6**	**14**	**12.4**							
**Radius**						**GL**	**Bp**	**Bd**								
**Haluza**	**G-10/15**	**1/05a**	**29**	**Byzantine**	**chicken**	**65.1**										
**Nizzana**	**G4/17**	**103**	**1218/5**	**Early Islamic**	**chicken**	**71.5**										
**SH**	**G87/15**	**401**	**4026**		**dog**			**25.8**								
**Nizzana**	**G4/17**	**105**	**1236/1**	**Early Islamic**	**gazelle**			**21**								

**Nizzana**	**G4/17**	**103**	**1177/4**	**Early Islamic**	**gazelle**			**22.1**								
**Nizzana**	**G4/17**	**103**	**1218/5**	**Early Islamic**	**gazelle**		**23.4**									
**Nizzana**	**G4/17**	**103**	**1177/4**	**Early Islamic**	**gazelle**		**23.9**									
**Nizzana**	**G4/17**	**103**	**1218/5**	**Early Islamic**	**gazelle**		**26.6**									
**Nizzana**	**G4/17**	**122**	**1426/1**	**Byzantine**	**goat**		**36.6**									
**Nizzana**	**G4/17**	**103**	**1177/4**	**Early Islamic**	**goat**			**30.9**								
**Nizzana**	**G4/17**	**103**	**1156/3**	**Early Islamic**	**goat**		**32**									
**Nizzana**	**G4/17**	**104**	**1210/2**	**Early Islamic**	**goat**		**34**									
**SH**	**G87/15**	**515**	**5115**		**goat**			**33.3**								
**SH**	**G87/15**	**410**	**4067**		**goat**		**38**									
**Nizzana**	**G4/17**	**105**	**1236/1**	**Early Islamic**	**pig**			**24.4**								
**Haluza**	**G10/15**	**7/01b**	**83**	**Byzantine**	**sheep**		**36.3**									
**SH**	**G4/16**	**168**	**1668**	**Byzantine**	**sheep**		**35.7**									
**Nizzana**	**G4/17**	**103**	**1135/2**	**Early Islamic**	**sheep**		**35.9**									
**Haluza**	**G-10/15**	**1/03b**	**15**	**Byzantine**	**shg**			**30.9**								
**Haluza**	**G-10/15**	**4/01d**	**11**	**Byzantine**	**shg**			**34.4**								
**Nizzana**	**G4/17**	**104**	**1210/2**	**Early Islamic**	**shg**			**25.6**								

**Nizzana**	**G4/17**	**103**	**1177/4**	**Early Islamic**	**shg**		**32.1**									
**Nizzana**	**G4/17**	**103**	**1177/4**	**Early Islamic**	**shg**		**34.2**									
**Nizzana**	**G4/17**	**107**	**1295**	**Early Islamic**	**shg**		**36.1**									
**SH**	**G87/15**	**854**	**8531**		**shg**			**27.1**								
**SH**	**G87/15**	**250**	**2501**		**shg**		**35.2**									
**Ovdat**	**A-3141**	**1122**	**126**	**L Roman/E Byzantine**	**shg**			**29.2**								
**Ovdat**	**A-3141**	**1122**	**116**	**L Roman/E Byzantine**	**shg**			**30.2**								
**Ovdat**	**A-3141**	**1122**	**126**	**L Roman/E Byzantine**	**shg**			**31.7**								
**Ovdat**	**A-3141**	**1801**	**305**	**L Roman/E Byzantine**	**shg**			**33**								
**Ovdat**	**A-3141**	**1143**	**171**	**L Roman/E Byzantine**	**shg**			**34.3**								
**Ovdat**	**A-3141**	**1142**	**161**	**L Roman/E Byzantine**	**shg**			**34.4**								
**Ovdat**	**A-3141**	**1202**	**40**	**L Roman/E Byzantine**	**shg**			**35.7**								
**Ovdat**	**A-3141**	**1202**	**43**	**L Roman/E Byzantine**	**shg**			**38.1**								
**Scapula**						**GLP**	**BG**									
**Nizzana**	**G4/17**	**213**	**2085/1**	**Byzantine**	**camel**	**110.9**										
**Nizzana**	**G4/17**	**103**	**1156/3**	**Early Islamic**	**fox**	**14.7**	**9**									
**Nizzana**	**G4/17**	**105**	**1236/1**	**Early Islamic**	**gazelle**	**25.7**										

**Nizzana**	**G4/17**	**105**	**1236/1**	**Early Islamic**	**gazelle**	**26**	**17.5**									
**Nizzana**	**G4/17**	**103**	**1177/4**	**Early Islamic**	**gazelle**	**27.5**	**18.8**									
**Haluza**	**G-10/15**	**4/04d**	**40**	**Byzantine**	**hare**	**16.1**										
**Haluza**	**G-10/15**	**1/06a**	**33**	**Byzantine**	**pig**	**29.9**										
**Nizzana**	**G4/17**	**103**	**1177/4**	**Early Islamic**	**pig**	**37.4**	**23.4**									
**Haluza**	**G10/15**	**7/01c**	**84**	**Byzantine**	**shg**	**33.4**										
**Haluza**	**G10/15**	**6/02d**	**81**	**Byzantine**	**shg**	**36.6**										
**Nizzana**	**G4/17**	**110**	**1326/1**	**Byzantine**	**shg**	**36.4**	**22.2**									
**Nizzana**	**G4/17**	**103**	**1121/1**	**Early Islamic**	**shg**	**33.1**	**23.5**									
**Nizzana**	**G4/17**	**103**	**1156/3**	**Early Islamic**	**shg**	**39.2**	**24.4**									
**Nizzana**	**G4/17**	**103**	**1177/4**	**Early Islamic**	**shg**	**40.8**	**25.6**									
**SH**	**G87/15**	**153**	**1593**	**Early Islamic**	**shg**	**30.2**	**21.5**									
**Ovdat**	**A-3141**	**704**	**84**	**L Roman/E Byzantine**	**equid**	**75**										
**Ovdat**	**A-3141**	**704**	**87**	**L Roman/E Byzantine**	**shg**	**32.1**										
**Ovdat**	**A-3141**	**1143**	**171**	**L Roman/E Byzantine**	**shg**	**34.5**										
**Ovdat**	**A-3141**	**1122**	**126**	**L Roman/E Byzantine**	**shg**	**34.5**										
**Ovdat**	**A-3141**	**1122**	**116**	**L Roman/E Byzantine**	**shg**	**35.1**	**21.2**									

**Ovdat**	**A-3141**	**704**	**95**	**L Roman/E Byzantine**	**shg**	**37.4**										
**Ovdat**	**A-3141**	**1122**	**116**	**L Roman/E Byzantine**	**shg**	**37.4**	**23.8**									
**Ovdat**	**A-3141**	**702**	**63**	**L Roman/E Byzantine**	**shg**	**41.4**										
**Ovdat**	**A-3141**	**1142**	**161**	**L Roman/E Byzantine**	**shg**	**42.4**										
**Ovdat**	**A-3141**	**702**	**53**	**L Roman/E Byzantine**	**shg**	**42.4**										
**Ovdat**	**A-3141**	**1122**	**116**	**L Roman/E Byzantine**	**shg**	**42.5**	**25.9**									
**Tibiotarsus**						**Bd**	**Dd**	**GL**								
**Haluza**	**G10/15**	**1/01e**	**100**	**Byzantine**	**chicken**	**10.7**										
**Haluza**	**G10/15**	**1/01e**	**100**	**Byzantine**	**chicken**	**11.5**										
**Nizzana**	**G4/17**	**121**	**1352/1**	**Byzantine**	**chicken**	**12.7**										
**Nizzana**	**G4/17**	**116**	**1533/1**	**Byzantine**	**chicken**	**13.3**										
**Nizzana**	**G4/17**	**103**	**1135/2**	**Early Islamic**	**chicken**	**10.9**										
**Nizzana**	**G4/17**	**103**	**1121/1**	**Early Islamic**	**chicken**	**12.1**										
**Nizzana**	**G4/17**	**103**	**1135/2**	**Early Islamic**	**chicken**	**12.5**										
**Nizzana**	**G4/17**	**103**	**1156/3**	**Early Islamic**	**chicken**	**12.7**										
**Nizzana**	**G4/17**	**103**	**1135/2**	**Early Islamic**	**chicken**	**12.9**	**13.6**									
**Nizzana**	**G4/17**	**103**	**1156/3**	**Early Islamic**	**chicken**	**13**										

**Nizzana**	**G4/17**	**103**	**1177/4**	**Early Islamic**	**chicken**	**13.1**										
**Nizzana**	**G4/17**	**103**	**1177/4**	**Early Islamic**	**chicken**	**14.6**										
**Nizzana**	**G4/17**	**103**	**1135/2**	**Early Islamic**	**pigeon**	**5.5**										
**Ovdat**	**A-3141**	**1202**	**43**	**L Roman/E Byzantine**	**?coot**	**8.6**										
**Ovdat**	**A-3141**	**1102**	**256**	**L Roman/E Byzantine**	**chicken**	**11.2**										
**Ovdat**	**A-3141**	**1122**	**116**	**L Roman/E Byzantine**	**chicken**	**11.5**										
**Ovdat**	**A-3141**	**1122**	**116**	**L Roman/E Byzantine**	**chicken**	**12.4**										
**Ovdat**	**A-3141**	**6362**	**711**	**L Roman/E Byzantine**	**chicken**	**10**		**101.4**								
**Ovdat**	**A-3141**	**1102**	**46**	**L Roman/E Byzantine**	**chicken**			**104.6**								
**Ovdat**	**A-3141**	**1122**	**116**	**L Roman/E Byzantine**	**chicken**	**12.9**		**126.1**								
**Tibia**						**Bd**	**Dd**									
**Nizzana**	**G4/17**	**105**	**1236/1**	**Early Islamic**	**gazelle**	**20.8**	**16.8**									
**Nizzana**	**G4/17**	**103**	**1177/4**	**Early Islamic**	**gazelle**	**21.3**	**17.6**									
**Nizzana**	**G4/17**	**105**	**1236/1**	**Early Islamic**	**gazelle**	**21**	**16.9**									
**Nizzana**	**G4/17**	**105**	**1236/1**	**Early Islamic**	**gazelle**	**22.3**	**17.9**									
**Nizzana**	**G4/17**	**103**	**1177/4**	**Early Islamic**	**gazelle**	**22**	**18.4**									
**Nizzana**	**G4/17**	**103**	**1218/5**	**Early Islamic**	**gazelle**	**23.8**	**18.4**									

Nizzana	G4/17	104	1210/2	Early Islamic	goat	29.5										
Haluza	G10/15	1/01e	100	Byzantine	shg	29.2	22.2									
Haluza	G-10/15	1/01a	9	Byzantine	shg	29.7	23.7									
Haluza	G-10/15	4/05c	47	Byzantine	shg	30.5	22.1									
Haluza	G-10/15	7/01b	83	Byzantine	shg	32.4	25.7									
Nizzana	G4/17	509	5103	Byzantine	shg	26.7	20.9									
Nizzana	G4/17	119	1398/4	Byzantine	shg	26	19.7									
Nizzana	G4/17	124	1418/1	Byzantine	shg	28.6	22.3									
Nizzana	G4/17	509	5103	Byzantine	shg	29	24.2									
Nizzana	G4/17	103	1177/4	Early Islamic	shg	23.5	19.2									
Nizzana	G4/17	101	1048/3	Early Islamic	shg	25.8	20.7									
Nizzana	G4/17	104	1210/2	Early Islamic	shg	26.5										
Nizzana	G4/17	103	1156/3	Early Islamic	shg	28.7	21.8									
Nizzana	G4/17	103	1156/3	Early Islamic	shg		21.3									
SH	G87/15	207	2033		shg	30.9	23									
SH	G87/15	410	4067		shg		26.2									
Ovdat	A-3141	1902	310	L Roman/E Byzantine	camel	82										

**Ovdat**	**A-3141**	**171**	**1143**	**L Roman/E Byzantine**	**deer**	**35.5**	**27.9**									
**Ovdat**	**A-3141**	**6362**	**711**	**L Roman/E Byzantine**	**donkey**	**63.1**	**44.8**									
**Ovdat**	**A-3141**	**"mezaveh"**		**L Roman/E Byzantine**	**gazelle**	**23**	**18.2**									
**Ovdat**	**A-3141**	**1102**	**46**	**L Roman/E Byzantine**	**shg**	**24.8**										
**Ovdat**	**A-3141**	**1122**	**126**	**L Roman/E Byzantine**	**shg**	**25.5**	**19.6**									
**Ovdat**	**A-3141**	**1302**	**290**	**L Roman/E Byzantine**	**shg**	**28.5**	**21**									
**Ovdat**	**A-3141**	**4042**	**638**	**L Roman/E Byzantine**	**shg**	**28.5**	**22.1**									
**Ovdat**	**A-3141**	**1302**	**296**	**L Roman/E Byzantine**	**shg**	**28**	**20.9**									
**Ovdat**	**A-3141**	**1122**	**126**	**L Roman/E Byzantine**	**shg**	**29.3**	**23.3**									
**Ovdat**	**A-3141**	**1121**	**100**	**L Roman/E Byzantine**	**shg**	**29.6**	**22.5**									
**Ovdat**	**A-3141**	**1302**	**290**	**L Roman/E Byzantine**	**shg**	**30.1**	**23.5**									
**Ovdat**	**A-3141**	**1302**	**290**	**L Roman/E Byzantine**	**shg**	**30.2**	**23.9**									
**Ovdat**	**A-3141**	**701**	**14**	**L Roman/E Byzantine**	**shg**	**30.3**	**23**									
**Ovdat**	**A-3141**	**702**	**63**	**L Roman/E Byzantine**	**shg**	**30.5**	**22.8**									
**Ovdat**	**A-3141**	**1101**	**7**	**L Roman/E Byzantine**	**shg**	**30.8**	**25.1**									
**Ovdat**	**A-3141**	**"mezaveh"**		**L Roman/E Byzantine**	**shg**	**31.4**	**24.3**									
**Ovdat**	**A-3141**	**4042**	**638**	**L Roman/E Byzantine**	**shg**	**33.4**	**26.1**									

**Ovdat**	**A-3141**	**1101**	**7**	**L Roman/E Byzantine**	**shg**	**33**	**25.1**									
**Ovdat**	**A-3141**	**1102**	**60**	**L Roman/E Byzantine**	**shg**	**34.7**	**27.6**									
**Tarsometatarsus**						**GL**	**Bd**									
**Haluza**	**G-10/15**	**7/04c**	**98**	**Byzantine**	**chicken**	**72**	**11.9**									
**Haluza**	**G-10/15**	**4/03b**	**26**	**Byzantine**	**chicken**	**83.5**	**13.4**									
**Haluza**	**G10/15**	**1/09b**	**53**	**Byzantine**	**chicken**	**85.1**	**13.7**									
**Nizzana**	**G4/17**	**107**	**1295**	**Early Islamic**	**chicken**		**13.1**									
**Nizzana**	**G4/17**	**103**	**1135/2**	**Early Islamic**	**chicken**	**96.9**										
**SH**	**G87/15**	**603**	**6006**		**chicken**	**75**	**12.9**									
**Ovdat**	**A-3141**	**702**	**63**	**L Roman/E Byzantine**	**chicken**		**13.5**									
**Ovdat**	**A-3141**	**1122**	**126**	**L Roman/E Byzantine**	**chicken**	**69.5**	**11.1**									
**Ovdat**	**A-3141**	**704**	**87**	**L Roman/E Byzantine**	**chicken**	**76.2**	**12.6**									
**Ovdat**	**A-3141**	**1142**	**149**	**L Roman/E Byzantine**	**chicken**	**79.5**										
**Ovdat**	**A-3141**	**701-702**	**room 3**	**L Roman/E Byzantine**	**chicken**	**80.5**	**12.8**									
**Ovdat**	**A-3141**	**1122**	**116**	**L Roman/E Byzantine**	**chicken**	**84.4**	**14.3**									
**Ulna**						**GL**										
**SH**	**G87/15**	**711**	**7033**		**pigeon**	**44.5**										

Supplement 4

	Nizzana				Haluza	Oboda	Shivta	
	L Byzantine		Early Islamic		L Byzantine	L Roman	L Byzantine	
Category	Garbage	Building	Garbage	Building	Garbage	Garbage	All	All
N(BSM)	208	21	773	11	546	938	259	134
Gnaw/digested	5	1	14		5	10	8	3
Weather	4		13			4	10	1
Butchered	26	1	97		74	53	27	3
Burnt 1, 2, 3	1, 15, 10		11, 53, 48		1, 15,38	3, 0, 2	4, 7, 18	13, 15, 64
N(fracture_morph)	60	8	269	7	80	69	90	58
Green fractures	24	6	157	3	43	42	41	27
Dry fractures	36	2	112	4	37	27	49	31
N(fragmentation)	17	0	82	5	101		21	3
broken epiphysis	3		7		9	8		1
complete epiphysis	1		3		7	15	5	
complete to distal shaft	5		31	2	12	42	9	2
complete to mid-shaft	4		26	1	23	57	4	
complete to proximal shaft			6		8	14	2	
complete long bone	4		9	2	42	9	1	

Supplement 5

		Nizzana		Haluza	Ovdat	Shivta	
Taxon	Element	L Byzantine	Early Islamic	L Byzantine	L Roman	L Byzantine	Early Islamic
Sh/G	Skull	1	3	2	7	2	1
	Teeth, upper*	18 (6)	28 (8)	49 (20)	N/A	13 (4)	2 (1)
	Teeth, lower*	16 (5)	27 (9)	53 (13)	104 (63)	11 (7)	3 (2)
	Scapula	5	9	13	33	1	3
	Humerus	6	11	12	42	4	2
	Radius	4	14	9	20	6	2
	Carpals		1	3	6		
	Pelvis	9	23	25	25	2	3
	Femur	2	10	7	17	5	2
	Tibia	6	14	9	19	6	1
	Astragalus	3	3	5	24	1	3
	Calcaneus	3	9	7	12	5	1
	Metapodials	12	28	46	34	11	4
	Phalanx 1	12	9	20	37	9	8
	Phalanx 2	1	3	20	5	4	7
	Phalanx 3	1	4	8	2	4	3
	Total	99	196	288	387	84	45
Pig	Skull	2	6		1		
	Teeth, upper*	2 (1)	6 (2)	2 (1)		2 (1)	
	Teeth, lower*	1 (1)	15 (2)	2 (1)		4 (2)	
	Scapula		6	4			
	Humerus	2	2	2			
	Radius		2	1		2	1
	Carpals						
	Pelvis		1	1		1	
	Femur						
	Tibia		1	3		2	

	Astragalus		3				
	Calcaneus			1			
	Metapodials	3	18	4		2	
	Phalanx 1	1	2	2		2	1
	Phalanx 2		5	1		1	
	Phalanx 3		2				
	Total	11	69	23	1	16	2
Gazelle	Skull		3				
	Teeth, upper*		4 (2)			1 (1)	
	Teeth, lower*	1 (1)	10 (7)				
	Scapula	1	4				
	Humerus	1	8				
	Radius		7				
	Carpals						
	Pelvis		2				1
	Femur	1	2	1			
	Tibia		10		1		
	Astragalus		5				1
	Calcaneus						
	Metapodials	1	10	1			1
	Phalanx 1	1	3				
	Phalanx 2	2	1				1
	Phalanx 3		1				
	Total	8	70	2	1	1	4

* Numbers in parentheses are minimum number of elements for the maxillae and mandibulae.

Supplement 6

Taxon & Element		Nizzana				Haluza		Avdat		Shivta			
		Byzantine		Early Islamic		Byzantine		L Roman		L Byzantine		Early Islamic	
Taxon	Element	Fused	Unfused	Fused	Unfused	Fused	Unfused	Fused	Unfused	Fused	Unfused	Fused	Unfused
Sh/G	Scapula	2		6	1	10	1	23	5	1		1	
	Humerus	3		4	3	8	1	34	7	4		1	
	Radius	1		1	3	1	3	10	10	1			1
	Femur			2	3	2	1	7	10		2		
	Tibia	2	4	5	5	5	4	18	1	3	3		1
	Metapodial	5	2	8	6	18	3	27	15	4			1
	Phalanx 1	8	2	4	4	14	2	27	10	4	2	4	2
	Total	21	8	30	25	58	15	146	58	17	7	6	5
Pig	Scapula			2	1	2	2						
	Humerus			1	1	1	1						
	Radius				1								
	Femur												
	Tibia						3				2		
	Metapodial		1	1	7		2						
	Phalanx 1		1		1	1	1				2		1
	Total		2	4	11	4	9				4		1
Gazelle	Scapula	1		4									
	Humerus	1		4									
	Radius			2	2								
	Femur	1			1		1						
	Tibia			9									
	Metapodial	1		4	3							1	
	Phalanx 1	1		3									
	Total	5		26	6		1					1	

Supplement 7

Nizzana	Young	Juvenile		Adult	
Byzantine	*1*	*2*	*3*	*4*	*5*
Sheep	1		1		
Goat	2		1	2	3
S+G+Sh/G*	3		2	2	3
Pig				1	
Gazelle			1		
EIS	*1*	*2*	*3*	*4*	*5*
Sheep	2		3	3	1
Goat			2	2	
S+G+Sh/G*	6		6	6	1
Pig	5	1		1	
Gazelle	3		3		1
Haluza					
Byzantine	*1*	*2*	*3*	*4*	*5*
Sheep	3	1		4	1
Goat	4	1		5	3
S+G+Sh/G*	11	2		12	6
Pig	1				
Avdat					
L Roman	*1*	*2*	*3*	*4*	*5*
Sheep	14	5	10	13	
Goat	6		1	9	
S+G+Sh/G*	22	9	14	23	
Shivta					
Byzantine	*1*	*2*	*3*	*4*	*5*
Sheep	4			1	
Goat		1		1	1
S+G+Sh/G*	4	2		2	1
Pig	2	1			
Gazelle					
